# Implantable Self‐Powered Systems for Electrical Stimulation Medical Devices

**DOI:** 10.1002/advs.202412044

**Published:** 2024-11-26

**Authors:** Xi Cui, Li Wu, Chao Zhang, Zhou Li

**Affiliations:** ^1^ Beijing Institute of Nanoenergy and Nanosystems Chinese Academy of Sciences Beijing 101400 China; ^2^ School of Biomedical Engineering Shenzhen Campus of Sun Yat‐Sen University Shenzhen 518107 China; ^3^ School of Nanoscience and Engineering Chinese Academy of Sciences Beijing 100049 China

**Keywords:** biological effects of electrical stimulation, biological tissue interface electrode, energy harvesting, implantable self‐powered systems, integration

## Abstract

With the integration of bioelectronics and materials science, implantable self‐powered systems for electrical stimulation medical devices have emerged as an innovative therapeutic approach, garnering significant attention in medical research. These devices achieve self‐powering through integrated energy conversion modules, such as triboelectric nanogenerators (TENGs) and piezoelectric nanogenerators (PENGs), significantly enhancing the portability and long‐term efficacy of therapeutic equipment. This review delves into the design strategies and clinical applications of implantable self‐powered systems, encompassing the design and optimization of energy harvesting modules, the selection and fabrication of adaptable electrode materials, innovations in systematic design strategies, and the extensive utilization of implantable self‐powered systems in biological therapies, including the treatment of neurological disorders, tissue regeneration engineering, drug delivery, and tumor therapy. Through a comprehensive analysis of the latest research progress, technical challenges, and future directions in these areas, this paper aims to provide valuable insights and inspiration for further research and clinical applications of implantable self‐powered systems.

## Graphical abstract

In this paper, the design strategy and clinical application of ISS are discussed in depth from four aspects: the design and optimization of the energy collection module, the selection and preparation of adaptive electrode materials, the innovation of system design strategy, and the biological effect of electrical stimulation of ISS.

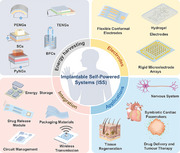

## 1 Introduction

Electricity plays a crucial role in human physiological activities, intricately intertwined with life processes. It has profound effects on various levels, including cells, tissues, organs, and even the entire organism.^[^ ^1^, ^2^ ^]^ Since ancient times, people have been fascinated by natural electrical phenomena. The connection between electricity and physiological activities can be traced back to the Roman Empire when people observed that electric eels could alleviate symptoms of certain ailments with their unique electric shocks.^[^ ^3^ ^]^ Notably, electrical stimulation, as an interventional modality, has demonstrated immense potential in the medical field. By applying external electrical signals to specific sites, it can modulate local or systemic electrophysiological activities, thereby achieving therapeutic goals and facilitating rehabilitation.^[^ ^4^, ^5^, ^6^ ^]^ For instance, in neuroscience, electrical stimulation is widely employed in the treatment of neurological and psychiatric disorders such as Parkinson's disease and depression.^[^ ^7^, ^8^, ^9^, ^10^ ^]^ In rehabilitation medicine, it aids in restoring and rebuilding muscle function.^[^ ^11^, ^12^ ^]^

However, the limitations of traditional electrical stimulation medical devices, particularly their reliance on external power sources or internal batteries, have hindered further advancements in miniaturization, portability, and long‐term monitoring.^[^ ^13^, ^14^ ^]^ To overcome these constraints, the emergence of self‐powered technology has revolutionized the landscape of electrical stimulation medical devices. This technology enables devices to directly harvest energy from the surrounding environment and convert it into electrical energy, achieving energy autonomy. Such a breakthrough not only opens up new possibilities for implantable applications of electrical stimulation medical devices but also significantly enhances their clinical feasibility.^[^ ^15^ ^]^

**Figure 1:**
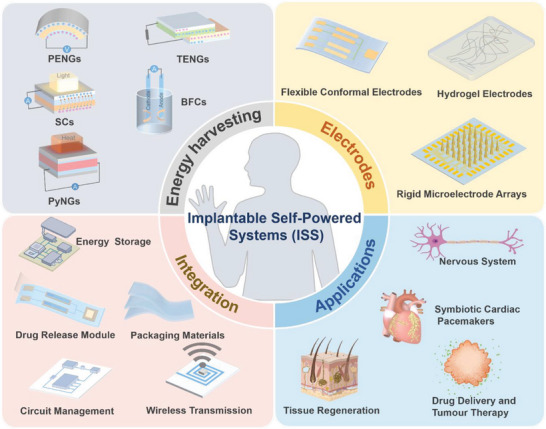
Overview of the design and innovation of advanced implantable self‐powered electronic medical devices based on energy sources, performance, and specific applications. Implantable self‐actuation systems can harvest mechanical, solar, thermal, and biological energy to power devices or directly stimulate tissues. This process requires the entire system to possess mechanical, electrical, and biological properties for long‐term stable operation in vivo. The application of implantable self‐energy systems in electronic medicine is mainly in the fields of cardiac pacing, neural regulation, tissue repair, and tumor treatment.

Therefore, this review aims to comprehensively review the design strategies and therapeutic applications of implantable self‐powered systems (ISS) for electrical stimulation medical devices. Four core aspects are delving: innovations in energy harvesting modules, optimization of electrode modules, advancements in systematic integration strategies, and the extensive applications of ISS in biotherapy (**Figure** **1**). Regarding the power supply module, which is crucial for achieving self‐powering in ISS, we highlight two advanced energy harvesting technologies: triboelectric nanogenerators (TENGs) and piezoelectric nanogenerators (PENGs). We also discuss the electrode module, which is critical for therapeutic efficacy as the direct point of electrical stimulation. Additionally, we analyze the strengths and weaknesses of rigid and flexible electrodes and explore the application of various materials in electrode fabrication. In terms of systematic integration, stable energy storage, circuit management modules, drug release modules, biocompatible packaging materials, and wireless network connectivity in ISS are introduced. Finally, we explore the extensive applications of ISS in biotherapy, including the treatment of neurological disorders, advancements in tissue engineering and regeneration, and innovative approaches in drug delivery and tumor therapy. We conclude by discussing and evaluating the challenges and prospects for the development of ISS in clinical electrical stimulation therapy.

## 2 Energy Harvesting Devices Design of ISS

**Figure 2:**
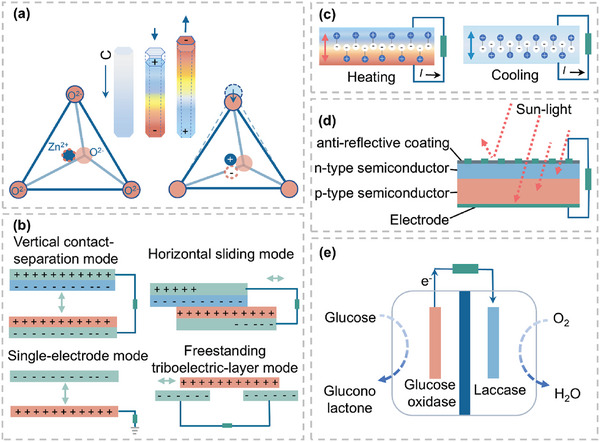
Energy harvesting devices design of self‐powered systems. a) Piezoelectric nanogenerators. b) Triboelectric nanogenerators. c) Pyroelectric nanogenerators. d) Solar cells. e) Biofuel cells.

Self‐powered technologies can convert mechanical, solar, thermal, and biochemical energy into electrical energy to power devices. To address the challenge of energy supply for implantable electronic medical devices, researchers have developed various nanogenerator structures, modes, and materials tailored to the energy requirements of different implanted sites and devices. Existing self‐powered technologies include PENG,^[^ ^16^ ^]^ TENG,^[^ ^17^ ^]^ solar cells (SCs),^[^ ^18^ ^]^ pyroelectric nanogenerators (PyNGs),^[^ ^19^ ^]^ and biofuel cells (BFCs)^[^ ^20^ ^]^ (**Figure** **2**).

### 2.1 Piezoelectric Nanogenerators

PENG is a type of nanogenerator that harnesses the piezoelectric effect to collect nanoscale mechanical energy (e.g., vibrations, compressions, flexions) and convert it into electrical energy. The piezoelectric effect refers to the phenomenon where internal electromotive force is generated in a material under mechanical stress. Taking zinc oxide (ZnO), a piezoelectric material with a lattice structure, as an example (Figure 2a), Zn^2^⁺ and O^2^^−^ ions are distributed in a tetrahedral configuration. When an external mechanical force is applied along the C‐axis, the original overlapping centers of positive and negative charges undergo a displacement, resulting in the formation of dipole moments. On a macroscopic scale, the superposition of these dipole moments creates a “piezoelectric potential” on the material's surface, which subsequently drives the flow of electrons in an external circuit. Consequently, PENG typically comprises an external load and piezoelectric materials capable of generating piezoelectric potentials.

Piezoelectric materials, including ZnO, zinc stannate (ZnSnO₃), barium titanate (BaTiO₃), lead zirconate titanate (PZT), polyvinylidene fluoride (PVDF), and its copolymer Poly(Vinylidenefluoride‐Tirfluoroethylene (P(VDF‐TrFE)), among others, exhibit unique properties suitable for various applications. Inorganic piezoelectrics, particularly, are renowned for their high piezoelectric coefficients and robust piezoelectric effects, rendering them prime candidates for piezoelectric PENGs. Dagdeviren et al. fabricated flexible PZT‐based PENGs capable of generating a short‐circuit current of 100 nA under cyclic bending, showcasing their potential.^[^ ^21^ ^]^ However, the inherent brittleness and biotoxicity of lead‐ and ceramic‐based materials hinder their widespread use in implantable electrical stimulation devices.

To address these limitations, researchers have explored the encapsulation of these materials with flexible and biocompatible substrates, such as polyimide and polydimethylsiloxane. In a pioneering study, Li et al. successfully implanted ZnO nanogenerators into living organisms in 2010, utilizing the electromechanical coupling and piezoelectric effects of ZnO nanowires to harness mechanical energy from heartbeats. This device, fixed on a polyimide substrate, generated an open‐circuit voltage of 3 mV and a short‐circuit current of 30 pA.^[^ ^22^, ^23^ ^]^

Subsequently, ZnO's piezoelectric properties and biocompatibility have been harnessed for powering implantable electronic devices. Jin et al. integrated ZnO nanowire arrays into soft materials, creating a flexible and biocompatible energy harvester. When implanted in the right ventricle of a pig, this device produced an open‐circuit voltage of 0.3 V, albeit insufficient for powering many implantable devices.^[^ ^24^ ^]^ Enhancing the piezoelectric properties of PVDF through material recombination and structural design is another avenue of research. Doping PVDF with ceramic fillers, carbon‐based materials, or metal nanoparticles increases the β‐phase content, thereby boosting its piezoelectric performance.^[^ ^25^, ^26^ ^]^ Additionally, multilayer polymer nanocomposites inhibit charge injection and migration, improving breakdown strength and energy density. Piezoelectric polymers are attractive for powering implantable electronic devices due to their controllable size, flexibility, and biocompatibility. Dong et al. further enhanced energy harvesting capabilities by combining porous P(VDF‐TrFE) films with a bending beam array, achieving a peak open‐circuit voltage of 4.5 V and a peak short‐circuit current of 200 nA.^[^ ^27^ ^]^ These advancements underscore the potential of piezoelectric materials in advancing the field of implantable energy harvesting and medical devices.

### 2.2 Triboelectric Nanogenerators

TENG, a nanogenerator devised by leveraging the triboelectric effect and electrostatic induction, is a prominent technology in energy harvesting.^[^ ^28^, ^29^, ^30^ ^]^ The triboelectric effect, a ubiquitous phenomenon in daily life, arises from the differing electron affinities among materials. When two dissimilar materials rub against each other or come into contact, charge transfer occurs between them. Upon separation induced by an external force, these materials' surfaces acquire opposite charges. Electrostatic induction, on the other hand, describes the process where a charged object, when in proximity to an uncharged conductor, induces an opposite charge distribution in the conductor (Figure 2b).^[^ ^31^ ^]^ TENG operates in four distinct modes: the vertical contact‐separation mode, horizontal sliding mode, single‐electrode mode, and freestanding triboelectric‐layer mode.

In the vertical contact‐separation mode, the TENG typically comprises three components: triboelectric layers, electrode layers, and an external load.^[^ ^32^ ^]^ During contact and subsequent separation, the two triboelectric layers convert mechanical energy into electrical energy. Their disparate electron‐capturing capabilities result in the formation of equal and opposite charges on their surfaces post‐contact. Upon separation, these static charges also part, creating a potential difference across the electrodes mounted on the layers' backsides. Upon connecting an external load, this potential difference propels charge flow, thereby generating a current. Under repetitive external forces, the TENG continuously undergoes contact‐separation cycles, sustaining a continuous current in the external load. This mode boasts a simple structural design, high instantaneous output power, and ease of multilayer integration, making it suitable for energy harvesters utilizing spacer structures, arch structures, and spring‐supported structures.^[^ ^33^, ^34^, ^35^ ^]^ The horizontal sliding mode employs two triboelectric layers and their respective back electrodes, positioned vertically.^[^ ^36^ ^]^ Horizontal movement alters the contact area between the layers, generating charges on their interfaces. These charges, in turn, induce charges on the back electrodes. Upon circuit connection, the induced potential difference prompts electron flow, generating a current. This mode excels in high instantaneous output power and offers structural versatility. The sliding structure's simplicity facilitates current augmentation through multi‐layering.^[^ ^37^ ^]^ Grid electrode structures optimize cost‐efficiency, enabling the enhancement of energy conversion efficiency by tuning sliding speed, electrode size, and dielectric layer thickness.^[^ ^38^ ^]^ The liquid metal structure stands out for its extensive contact area and high energy conversion efficiency.^[^ ^39^ ^]^ The single‐electrode mode involves two dielectric layers, with one electrode grounded. As the dielectric layers' proximity varies, the grounded electrode's local electric field distribution shifts, prompting electron flow between the electrode and ground, thus generating a current in the external load. Despite its relatively lower output, this mode's broad applicability stems from the absence of a connecting wire on one friction surface. It finds use in harnessing micro‐winds, raindrops, and other sources of mechanical energy, as well as in sensors for touch, speed, angle, pressure, and human health monitoring. The freestanding triboelectric layer mode arranges two electrodes horizontally beneath the dielectric layer, connected via a load and wires.^[^ ^40^ ^]^ Dielectric layer movement creates an uneven charge distribution between the electrodes, prompting positive charge transfer between electrodes and the load to equalize the potential distribution. Repetitive motion of the dielectric layer augments energy output efficiency. Notably, direct contact between the dielectric layer and electrodes is unnecessary, mitigating material wear and enhancing TENG durability. This mode's versatility extends to rotary, grid‐array, and sliding‐type energy harvesters, as well as blue ocean energy harvesting applications.^[^ ^41^ ^]^

In 2012, Professor Zhonglin Wang designed the first TENG based on a double‐layer stacked structure using Kapton and Polyethylene terephthalate (PET)films, which achieved an open‐circuit voltage of 3.3 V and a short‐circuit current of 0.6 µA, with a power density of 10.4 mW·cm⁻^3^. This breakthrough opened up new avenues for energy harvesting.^[^ ^17^, ^42^ ^]^ Due to its simplicity in structural design and fabrication, convenient energy harvesting capabilities, and high output efficiency, TENGs with high voltage and low current output are particularly well‐suited for electrical stimulation in biological regulation. These devices can effectively conduct cell regulation and research without compromising normal cellular function. The main factors affecting the output power of the friction nanogenerator are the contact area and the charge density of the material surface. Ouyang et al. developed a symbiotic cardiac pacemaker by using a nanostructured polytetrafluoroethylene (PTFE) as the triboelectric layer for the device in combination with a commercial pacemaker.^[^ ^43^ ^]^ This device, featuring a core‐shell and keel structure design, significantly enhanced stability and output performance. It was able to charge a 100 µF capacitor to 3.55 V using mechanical energy from heartbeats over 190 min. Liu et al. proposed a self‐powered, implantable cardiac pacemaker with a unique capsule structure. Employing spherical particles of polyoxymethylene (POM) and PTFE as triboelectric layers, the POM particles periodically roll over the curved PTFE surface during heartbeats. The electret properties of PTFE allow for substantial charge retention on its surface. When implanted in adult pigs, this device achieved a charge transfer of 8.5 nC per heartbeat. At a load resistance of 100 MΩ, the maximum power density reached 2200 mW m^−^^3^.^[^ ^44^ ^]^

### 2.3 Pyroelectric Nanogenerators

Pyroelectric nanogenerators (PyNGs), as an energy harvester, transform temperature fluctuation into electrical energy through the pyroelectric effect, which encompasses the spontaneous polarization exhibited by pyroelectric materials.^[^ ^45^ ^]^ In a state of temperature equilibrium, the thermally induced electric dipoles in the material undergo a certain degree of random motion along their respective symmetry axes, and the overall average strength of the material's spontaneous polarization remains unchanged, thus no current is generated. As the temperature increases, the motion of the electric dipoles intensifies, leading to a decrease in the material's overall spontaneous polarization. This reduction in polarization results in a decrease in the amount of induced charge on the electrodes, generating a current when connected to an external load. Conversely, a decrease in temperature slows down the motion of the electric dipoles, enhancing the spontaneous polarization and increasing the charge accumulation on the electrodes, which in turn results in a reverse current flow through the external load (Figure 2c).^[^ ^46^ ^]^

Pyroelectric materials are mainly divided into ceramics, single crystals, inorganic thin films, and composite materials. Commonly used pyroelectric materials include PZT, barium titanate (BTO), ZnO, and PVDF. In 2012, Zhang and his team groundbreakingly demonstrated that temporal temperature fluctuations could elicit spontaneous polarization in PZT thin films. Specifically, with a temperature variation of 45 K, a PZT thin film‐based pyroelectric nanogenerator achieved an output voltage of up to 22 V.^[^ ^47^ ^]^ Notably, the crystal structure of BTO thin films undergoes a phase transition from cubic to orthorhombic during the processing from powder to ceramic form. This transformation enhances the material's spontaneous polarization properties. Leveraging this property, Song et al. devised a thermoelectric‐piezoelectric hybrid sensor array composed of BTO ceramics, encapsulated in a PDMS package for flexibility. This array boasts a sensitivity of ≈0.048 V °C^−1^, enabling simultaneous detection of temperature and pressure within a device.^[^ ^48^ ^]^ ZnO, in addition to its excellent piezoelectric properties, exhibits spontaneous polarization stemming from time‐dependent temperature variations within its internal crystal structure.^[^ ^45^ ^]^ Peng et al. capitalized on this characteristic, designing a high‐performance UV photodetector based on the thermoluminescence electron effect of ZnO/perovskite heterojunctions. This design exhibited a remarkable increase in UV photocurrent response, by 174.1% at 77 K and 28.7% at 300 K.^[^ ^49^ ^]^ In general, PyNGs embody advantages such as high durability, robust environmental adaptability, and flexibility. Their output performance is inherently tied to the pyroelectric coefficient of the material employed and the magnitude of temperature changes. Consequently, PyNGs have widespread applications in domains including fire alarms, thermal sensing, thermal imaging, and pollution monitoring.^[^ ^50^, ^51^, ^52^ ^]^

In recent years, with the development of nanomaterials, pyroelectric biomaterials have gradually attracted attention. Research has shown that photothermal therapy can induce effective immunogenic cell death.^[^ ^53^ ^]^ Although heated cancer cells can develop thermal resistance, pyroelectric materials with high robust reactive oxygen species (ROS) generation ability provide an effective solution to overcome this drawback. Upon the application of a specific temperature change, the charges liberated by pyroelectric materials interact with the oxygen present in the tumor microenvironment, leading to the copious generation of ROS. This process, in turn, consumes heat shock proteins and diminishes the thermal resilience of tumor cells.^[^ ^54^ ^]^ Li et al. designed a pyroelectric nanogenerator based on a high‐performance organic pyroelectric nanoplatform (^t^Bu‐TPAD‐BF_2_). Upon endocytosis by tumor cells, these nanoparticles, when exposed to near‐infrared light, efficiently generated ROS, resulting in the marked suppression of both primary and metastatic tumor growth and dissemination.^[^ ^55^ ^]^ The human body, as a highly sophisticated and stable biological system, undergoes rigorous physiological regulation to maintain its internal temperature within a relatively narrow and stable range of fluctuation. This implies that, despite the potential existence of a certain degree of temperature difference between the human surface and the external environment, the temperature disparities among various tissues and organs within the body are extremely minimal, falling far short of the temperature difference threshold required for the operation of PyNGs.

### 2.4 Solar Cells

Solar cells (SCs) are energy harvesters that convert light energy into electrical energy through the photovoltaic effect exhibited by semiconductors (Figure 2d). P‐type and N‐type semiconductors can be formed by the incorporation of phosphorus and boron atoms into semiconductor silicon, respectively. The combination of the two semiconductors can form a P‐N junction. When sunlight illuminates the P‐N junction within the semiconductor, a photo‐generated electric field, which is oriented opposite to the barrier's inherent direction, is created. When the external circuit is connected, a current can be generated.^[^ ^56^ ^]^ With the development of photovoltaic technology, researchers have gradually developed various types of SCs, including monocrystalline silicon, polycrystalline silicon, perovskite, and lead‐free perovskite SCs.^[^ ^57^ ^]^

Due to their permanence, flexibility, and cleanliness, SCs are widely used for power supply in fields such as communications, transportation, and meteorology. In 2004, Laube et al. implanted an artificial crystalline lens containing an array of SCs and light‐emitting diodes (LED) into rabbits. Long‐term testing showed that powering implanted electronic devices using SCs is feasible. However, due to contact defects between the solar cell and the LED, the final service life of the system ranges from 14 days to 7 months.^[^ ^58^ ^]^ Other researchers implanted flexible SCs subcutaneously in rats for energy harvesting. Because of the influence of energy conversion efficiency, the device's output power was 647 µW.^[^ ^59^ ^]^ With the development of organic SCs, energy conversion efficiency has significantly improved. Lv et al. used non‐fullerene acceptor materials as light‐harvesting materials in SCs, and the prepared flexible fiber‐shaped SCs achieved a power conversion efficiency of over 9% under standard 1.5 G irradiation.^[^ ^60^ ^]^ Overall, using a wide‐bandgap polymer donor (PM6), a narrow‐bandgap non‐fullerene acceptor (Y6), and phenyl‐C71‐butyric‐acid‐methyl ester(PC71BM)as a third component to adjust the optical absorption and morphology of the blend film is an effective way to improve organic SCs' performance in ternary heterojunctions.^[^ ^61^ ^]^ Despite demonstrating certain potential in implantable medical devices, SCs face numerous challenges. First, subcutaneous light utilization is a core issue, as light undergoes scattering and absorption upon penetrating skin, leading to a rapid decrease in light intensity, which renders implantation in deeper tissues virtually impossible. Furthermore, the biocompatibility of materials used in solar cells poses a significant challenge.

### 2.5 Biofuel Cells

A biofuel cell is an energy harvester that generates electrical energy by obtaining biochemical energy from living organisms or biological environments.^[^ ^37^ ^]^ A suitable biofuel and catalyst are positioned on the anode side of the biofuel cell, while oxygen is employed for reduction at the cathode to generate electrical energy (Figure 2e). Enzymatic biofuel cells (BFCs), which use glucose oxidase as the anode catalyst and laccase as the cathode catalyst to harvest energy through redox reactions, are considered one of the reliable power sources for implantable electronic devices. The electrochemical reaction process is as follows:^[^ ^38^ ^]^

(1) $$\;\mathrm{Anode}:\;\;C_{6}H_{12}O_{6}+2OH^{-}\to C_{6}H_{12}O_{7}+H_{2}O+2e^{-}$$

(2) $$\mathrm{Cathode}:\;\;\frac{1}{2}O_{2}+H_{2}O+2e^{-}\to 2OH^{-}$$

(3) $$\mathrm{Entirety}:\;C_{6}H_{12}O_{6}+\frac{1}{2}O_{2}\to C_{6}H_{12}O_{7}$$

Energy harvesting has already been successfully achieved in various plants and animals, including cacti, cockroaches, snails, and lobsters.^[^ ^62^ ^]^ In another study, a glucose biofuel cell based on carbon nanotube/enzyme electrodes was implanted in the abdominal cavity of a rat, could produce an open‐circuit voltage of 0.57 V and output power of 38.7 µW, and it operated in vivo for up to 110 days.^[^ ^63^ ^]^ Increasing the enzyme loading area and the electroactive surface area of the electrodes is an effective way to improve power output density. Wang et al. improved enzyme immobilization by designing a porous antifouling interface, and achieved a maximum output power of 76.6 mW in rabbits.^[^ ^64^ ^]^ However, excessive volume is not conducive to implantation in the body and is more likely to cause an immune response. Inorganic nanomaterials, used as catalysts, demonstrate higher catalytic efficiency, better durability and stability, and greater tunability and multifunctionality in BFCs compared to traditional catalysts. These characteristics enable it to effectively reduce the device volume and improve the power density. Gao et al. designed a flexible fiber biofuel cell based on carbon nanotubes that resist nonspecific protein adsorption, which generated a power density of 4.4 µW cm⁻^2^ in the brains of mice, its power density decreasing to 2.5 µW cm⁻^2^ after one month.^[^ ^65^ ^]^ Guan et al. developed an implantable biofuel cell based on activated carbon nanotubes and N‐hydroxysuccinimide, which stabilized free glucose oxidase by forming amide bonds, enabling rapid electron transfer between the enzyme and substrate. This device achieved an open‐circuit voltage of 0.575 V and a power density of 57 µW cm⁻^2^ in a 5 mM glucose solution.^[^ ^66^ ^]^ However, the operating voltage of implantable electronic medical devices is typically in the range of 2–3 V, and the thermodynamic constraints imposed by the redox potential in BFCs result in very limited output voltage, insufficient to power most implantable devices. BFCs can be classified into microbial fuel cells, enzymatic fuel cells, and photocatalytic fuel cells, depending on the type of catalyst used. The applications of different types of BFCs also vary, such as microbial fuel cells and photocatalytic fuel cells being widely used for wastewater treatment and power generation,^[^ ^67^, ^68^ ^]^ while enzymatic fuel cells are commonly used as biosensors.^[^ ^69^ ^]^ Among these, the enzyme loading capacity and electron transfer rate are key factors affecting the performance of the cells. The enzyme loading capacity and electron transfer rate of BFCs are susceptible to the influence of complex environments within biological organisms, such as temperature variations, pH fluctuations, and interference from biomolecules. Researchers usually adopt structural design and material synthesis methods to improve cell performance, however, challenges such as low output power and short lifespan remain difficult to overcome.

In summary, ISS based nanogenerators can harvest energy from the body and surrounding environment to power electronic devices, showing great potential in the design and application of implantable electronic medical devices. As a result, numerous researchers have explored different power supply methods, such as using SCs to power commercial pacemakers,^[^ ^70^ ^]^ harvesting energy from heartbeats via oscillators to power implantable cardiac electronic medical devices,^[^ ^71^ ^]^ and utilizing the electrical energy generated by the redox reaction of glucose in the body,^[^ ^72^ ^]^ to power implantable cardiac electronic medical devices. However, each method has its scope of applicability and limitations, and not all self‐powered technologies are suitable for powering and sensing in implantable electronic medical devices. For example, PyNGs require a large temperature difference to generate charge transfer, making them unsuitable for in vivo use due to the minimal temperature fluctuations within the human body,^[^ ^73^ ^]^ the energy conversion efficiency of SCs is significantly affected by implantation depth, presenting challenges in terms of subcutaneous light utilization, miniaturization, biocompatibility, and comfort,^[^ ^74^ ^]^ and while glucose BFCs can meet the demands of implantable electronic medical devices, it stills suffer from low output power and poor stability.^[^ ^75^ ^]^ On the other hand, TENGs and PENGs possess advantages such as adjustable shapes, controllable parameters, small size, good biocompatibility, high output, high sensitivity, low cost, and ease of fabrication. Many researchers have utilized these excellent properties in various fields, including energy harvesting from mechanical sources, wind power, and ocean energy,^[^ ^76^ ^]^ sensors for motion, speed, and physiological signals,^[^ ^77^ ^]^ and stimulators for tissue repair, cell regulation, and cardiac pacing.^[^ ^78^ ^]^ Next, we will mainly introduce the application of self‐powered systems based on TENG and PENG in electrical stimulation therapy.

## 3 Electrode Design for Bio‐Tissue Interface of ISS

With the development of electronics and bioelectronics, the approach to treating chronic diseases has gradually expanded from traditional drug therapies to electrical stimulation. Research has shown that applying external currents or electric fields can significantly activate or inhibit the physiological activities of molecules, cells, tissues, and organs. Currently, FDA‐approved implantable electronic medical devices, such as pacemakers, deep brain stimulators, cochlear implants, and vagus nerve stimulators, function by delivering electrons from an interface electrode to the target tissue. In the process, electrons act as charge carriers in the metal electrodes, while electrolytes serve as carriers in the tissues. Due to different carriers, and interactions between electrodes and tissue interfaces. Depending on the characteristics of the electrode material, this interaction can be divided into capacitive charge injection and Faradaic charge injection. In terms of capacitive charge injection, ions of opposite polarity are attracted, forming a compact layer of charges proximal to the electrode and a diffuse layer at the tissue interface. This arrangement constitutes a double electric layer, imparting capacitive properties to the electrode‐tissue interface. Consequently, when an external voltage is imposed, the double layer undergoes charging and discharging cycles, enabling charge transfer between the electrode and the electrolyte. Notably, during charge injection, the electrode undergoes no redox reactions, thereby minimizing corrosion and degradation, rendering it particularly apt for use as a stimulating electrode. For faradaic charge injection, which relies on redox reactions at the electrode‐electrolyte interface. For instance, during oxidation, a platinum electrode relinquishes electrons, resulting in the formation of platinum ions that subsequently exchange with counter‐ions in the electrolyte, generating a current. In Faradaic charge injection, the ideal electrochemical reaction should be reversible, ensuring that the electrode material remains undegraded during oxidation‐reduction cycles. Such electrodes, composed of materials capable of reversible redox reactions, are preferred for recording purposes due to their frequency‐independent impedance, which mitigates filtering effects across various signal frequencies. Furthermore, Faradaic charge injection typically offers a high charge injection capacity, making it suitable for electrical stimulation applications. However, in practical scenarios, certain reactions may prove irreversible, potentially leading to electrode material corrosion or degradation. Ultimately, the selection of electrode material and its structural design are crucial determinants of the performance and long‐term stability of implantable electronic medical devices within the human body. Considering the diverse characteristics, requirements, and constraints of different tissues, a tailored approach to electrode design is essential for optimal device functionality and biocompatibility.

Early on, inert metals including platinum, gold, tungsten, and their alloys were extensively employed in the design and fabrication of neural electrodes, attributed to their stability in chemical properties, outstanding conductivity, and commendable biocompatibility within biological milieus.^[^ ^79^ ^]^ Given the distinct functions and roles of various brain regions and nuclei, these metallic electrodes are frequently tailored as single or multiplexed micro‐wires during stimulation and recording procedures, aiming to enhance localization precision and signal acquisition quality. Their diameters span from 2000 to 20 µm, and at a frequency of 1 kHz, the impedance of conventional metal electrodes typically falls within the range of 1 kΩ to 1 MΩ.^[^ ^80^ ^]^

Nonetheless, limitations persist, such as low interface capacitance and constrained charge injection capacity. Platinum electrodes or iridium oxide electrodes have demonstrated enhanced performance.^[^ ^81^ ^]^ It is worth noting that semiconductors have shown unique advantages in specific applications for both intrinsic and heterojunction neural interfaces. Inorganic semiconductor electrodes (silicon) are widely used because of their high electron mobility and low operating voltage despite their hard material.^[^ ^82^ ^]^ Organic semiconductors are soft and have good biocompatibility, typically providing a more conformal interface with target tissues.^[^ ^83^ ^]^ However, it is noteworthy that organic semiconductor materials may face challenges such as degradation, oxidation, or chemical changes during long‐term implantation, affecting their long‐term stability and performance.

**Figure 3:**
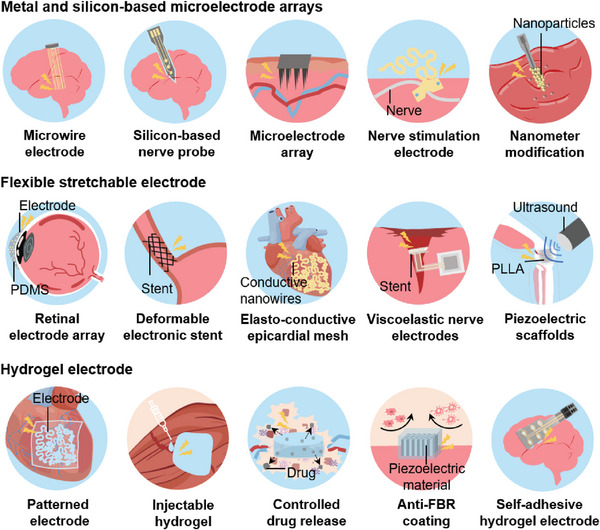
Types of implantable biotissue interface electrodes. a) Rigid microelectrode arrays. b) Flexible conformal electrodes. c) Hydrogel electrodes.

Over the past few decades, significant progress has been made by numerous researchers in the flexibility and stretchability of traditional metal electrodes. For instance, metal materials have been made into thin films, fibers, or composites through nanotechnology to achieve conformal contact with tissues. However, these designs do not change the intrinsic characteristics of the materials. In the process of tissue interaction, there are still some limitations such as interfacial interaction leading to increased interfacial impedance and reduced charge injection capacity. In addition, after the electrode is implanted into the body, acute inflammation will occur rapidly, and immune cells such as white blood cells and macrophages will gather around the implant. With the passage of time, it will gradually turn to chronic inflammation and fibrosis, which will affect the normal work of implantable electronic medical devices.^[^ ^84^ ^]^ Hydrogels, with their high‐water content, adjustable modulus, and multifunctional capabilities, have become very promising candidates for electrode materials. Therefore, when designing electrodes for bio‐tissue interfaces, we must take into account not only the physical properties of the electrodes, including interface impedance, charge injection capacity, surface morphology, and Young's modulus but also their biocompatibility and the intricacy of biological tissues. Next, we will discuss the electrode design for ISS used in bio‐tissue interface from the perspectives of rigid microelectrode arrays, flexible conformal electrodes, and hydrogel electrodes (**Figure** **3**).

### 3.1 Rigid Microelectrode Arrays

In the collection and modulation of neural signals, there are two main methods: one is non‐invasive electrode collection based on surface electromyography (EMG) and electrocorticography (ECoG), and the other is invasive electrode collection based on single‐electrode recordings and electrode arrays. Due to the brain tissue acting as a natural low‐pass filter, electrocorticography from the scalp or cortical electroencephalography from the dura mater can only capture low‐frequency electrical signals, making it difficult to detect high‐frequency spike discharges from individual neurons. Therefore, invasive electrode designs are required to record high‐frequency spike discharges from individual neurons or specific deep nuclei. In the early stages of neural stimulation and recording, metal electrodes were commonly chosen as tissue interface electrodes due to their high conductivity, good stability, and simple fabrication process. However, metal electrodes can exhibit double‐layer effects or electrochemical reactions at the interface with biological tissue, leading to increased interface impedance. To reduce impedance and enhance charge injection capacity, surface modification of the electrode interface is an important approach. The iridium oxide film sputtered on the Pt electrode of the microelectrode array showed a 10 fold lower impedance at 1 kHz compared to the Pt electrode.^[^ ^85^ ^]^ Depositing metal clusters on the surface of Ag electrodes can create nanozyme electrodes, which have an impedance 26 times lower than that of metal electrodes due to the presence of both high‐speed electron and ion currents at the electrode‐tissue interface. Additionally, nanozyme electrodes possess antioxidant and multi‐enzyme mimicking properties, which can reduce tissue damage caused by inflammatory responses.^[^ ^86^ ^]^ Surface modifications can induce changes in electrostatic surface charges; the 3,4‐ethylenedioxythiophene (PEDOT) coating is also a common method to reduce impedance.^[^ ^87^ ^]^

With the advancement of micro‐ and nano‐fabrication technologies, silicon‐based microelectrode arrays have gradually been used in the design of neural electrodes (Figure 3a), such as the Michigan probe and Utah electrode array.^[^ ^88^, ^89^ ^]^ The Michigan‐style silicon‐based probes can be designed with multiple electrode arrays along the plane of the shaft, allowing for precise control of implantation depth and minimizing intrusion into brain tissue, while also enabling highly selective recording and stimulation of brain tissue.^[^ ^90^ ^]^ In 2006, Hochberg and colleagues successfully implanted a Utah electrode array with 96 channels into the M1 area of a quadriplegic patient, enabling them to control a mechanical hand using neural signals.^[^ ^91^ ^]^ Compared to traditional metal‐based probes, complementary metal‐oxide‐semiconductor (CMOS) technology enables multiplexed, high‐density integration on silicon‐based electrodes. Using this technology, the neuropixels electrode has integrated 960 recording electrodes on a probe that is 10 mm long and 70 µm wide, with 384 independent recording channels that can be addressed among the electrodes, achieving high‐throughput recording in the mouse brain.^[^ ^92^ ^]^ In another study, Neuropixel electrodes combined with the advantages of Utah electrodes were arranged in a 4 × 4 configuration to form a 3D silicon probe array, expanding the sampling volume laterally.^[^ ^93^ ^]^

For the stimulation of muscles, eyes, and other tissues, the structure of the electrodes is also designed according to the curvature of the tissues. In muscle electrical stimulation, the cathode and anode are usually spaced a certain distance apart to induce muscle contraction.^[^ ^94^ ^]^ The electrode array for artificial retinas requires complex biomimetic structural design, using flexible polymers as encapsulation materials to prevent damage to nerves from the edges of the electrodes.^[^ ^95^ ^]^ Although rigid electrodes have made significant progress in terms of interface impedance, charge injection capacity, and integration, the issue of tissue damage caused by rigid probes has not yet been resolved.

### 3.2 Flexible Conformal Electrodes

**Figure 4:**
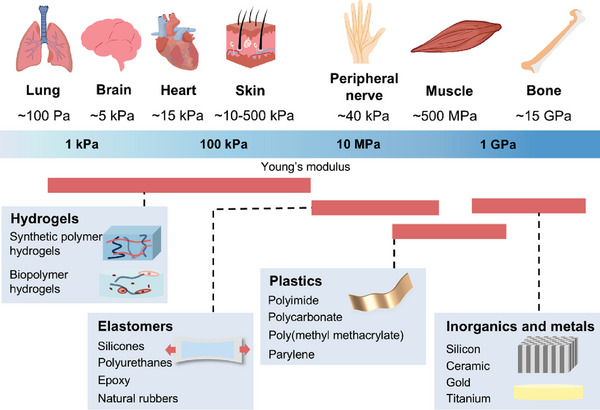
Young's modulus of organs and materials for ISS.

Young's modulus of traditional metal electrodes and some rigid materials (such as silicon) is typically much higher than that of biological tissues. For instance, Young's modulus of metals usually ranges from tens to hundreds of GPa, while Young's modulus of most soft tissues (such as brain and muscle) ranges from hundreds of Pa to several MPa (**Figure** **4**). When rigid electrodes are implanted into soft biological tissue, mechanical stress concentrates at the electrode‐tissue interface. As the tissue moves (e.g., due to breathing, heartbeat, brain expansion, and contraction), this stress can lead to local tissue deformation, damage, or inflammatory responses. The mismatch in stiffness can lead to non‐conformal contact between the interface electrodes and the tissue, causing the implant to shift position and delaminate. These shifts and delaminations, in turn, can result in chronic inflammation and tissue fibrosis, which increase interface impedance and degrade the quality of electrical signal transmission.

Flexible conformal electrodes, constructed from materials possessing a low elastic modulus, such as organic semiconductors and conductive polymers, offer a viable solution to address the aforementioned challenges. Additionally, by designing the geometry of metal electrodes (e.g., serpentine designs, island‐bridge designs), the bending stiffness of the material can be reduced, making it stretchable.^[^ ^96^ ^]^ By employing these strategies, organic and composite materials can be designed into electrodes that better match the modulus and morphology of tissues. Notable examples encompass artificial retinal prostheses, conformal cardiac electrodes, electrode patches tailored for the internal surfaces of organs, and neural scaffolds, as depicted in Figure 3b. In the context of bioelectronic medical devices, flexibility is typically defined by materials possessing a low Young's modulus, specifically below several megapascals, indicating their capacity to undergo bending and deformation in response to external forces. Besides Young's modulus, flexural rigidity can also quantify a material's ability to resist bending deformation under external forces. Studies have shown that devices with lower flexural rigidity can reduce the induction of immune responses.^[^ ^97^ ^]^ Since flexural rigidity is proportional to the cube of the material's thickness, ultra‐thin devices with low flexural rigidity can more effectively conform to curved organs or tissues.^[^ ^98^ ^]^ The surface of the brain is very wrinkled.

To ensure tighter coupling between electrodes and tissue, microneedle electrode arrays are fabricated on flexible mesh film substrates. The microneedle electrodes provide a certain degree of rigidity, while the flexible substrate can fully bend to conform to the brain surface.^[^ ^99^ ^]^ Polyimide (PI) has good biocompatibility and stability, and flexible tissue electrode interfaces based on PI are widely used as a less invasive approach.^[^ ^100^ ^]^ By patterning the PI substrate through photolithography and then sputtering platinum on its surface to serve as electrodes, these neural probes have exhibited good biocompatibility and stability when implanted in rats for 30 days.^[^ ^101^ ^]^ Structural design and patterning of metal electrodes can enhance flexibility compared to traditional metals by strategic design and patterning. For example, a serpentine‐designed electrode embedded in an elastomer forms a mesh that can perfectly wrap around the heart.^[^ ^102^ ^]^ This design achieves conformal contact between the electrodes and the heart while significantly increasing the electrode's surface area, allowing more efficient transmission of current to the tissue.

Carbon‐based nanomaterials, such as graphene, carbon nanotubes (CNTs), and fullerenes (C60), are generally considered to have excellent mechanical properties, including high strength, high conductivity, and flexibility. Although these materials exhibit extremely high strength and rigidity in single nanostructures, they can exhibit some flexibility in the form of films, fibers, or composites. Studies have shown that composite films made by mixing silver nanowires and carbon nanotube solutions have excellent electrochemical properties.^[^ ^103^ ^]^ Additionally, conductive polymer materials and elastomers with moduli similar to those of tissues can improve interfacial mechanical compatibility. By combining polymers (such as PDMS) with highly conductive materials (such as metal nanoparticles or carbon nanotubes), sufficient conductivity can be provided while retaining flexibility. By mixing Carbon Nanotube/Reduced Graphene Oxide (CNT/rGO) networks with PDMS, this simple method not only reduces the aggregation of carbon nanomaterials but also effectively minimizes the modulus mismatch between electrodes and tissues, improving interfacial mechanical compatibility.^[^ ^104^ ^]^ Other studies have incorporated carbon nanotubes into polymers such as polycaprolactone (PCL),^[^ ^105^ ^]^ poly‐L‐lactic acid (PLLA),^[^ ^106^ ^]^ and silk protein,^[^ ^107^ ^]^ which also ensure excellent conductivity, biocompatibility, and conformability.

PEDOT, as a type of polythiophene and its derivatives, is an excellent conductive polymer. When doped with poly (styrene sulfonate) (PSS), it allows the long chains of PEDOT to open up more and be less likely to aggregate. This also changes the valence state of PEDOT, improving its conductivity.^[^ ^108^ ^]^ PEDOT:PSS as an electrode modification layer can significantly improve charge injection capabilities.^[^ ^109^ ^]^ In one study, PEDOT:PSS as an electrode layer for a retinal prosthesis showed excellent performance.^[^ ^110^ ^]^ However, there are challenges due to the mismatch in mechanical properties and chemical differences between stretchable films and flexible conductive composites, which can limit the adhesion between conductive composites and the substrate electrodes.

### 3.3 Hydrogel Electrodes

To ensure that an electronic medical device operates effectively in internal tissues or organs over time, it is usually implanted in the body and connected to the tissues via electrode interfaces. Implants (such as metals, electrodes, stents, prostheses, etc.) can trigger immune responses from the body. Although advancements in nanomaterials and polymers have significantly reduced the Young's modulus of tissue interface electrodes, long‐term implantation can still cause discomfort and even reduce the performance of the implanted devices. Hydrogels are 3D structures formed by hydrophilic polymer chains that can absorb large amounts of water, giving them mechanical properties similar to biological tissues.^[^ ^111^, ^112^ ^]^ Generally, the Young's modulus of hydrogels ranges from several kilopascals to several megapascals, similar to the Young's modulus of many biological tissues (Figure 4), enabling good conformal contact with target tissues. By incorporating conductive polymers (such as polypyrrole, polyaniline, and PEDOT) or nanomaterials (such as graphene, and carbon nanotubes), the conductivity of hydrogel electrodes can be significantly enhanced.^[^ ^113^, ^114^, ^115^ ^]^ These additives can also improve the mechanical strength and electrochemical stability of hydrogels. As a result, hydrogel electrodes have been widely used in various implantable applications (Figure 3c).

In neural electrical stimulation, stable contact between electrodes and tissues can effectively reduce noise interference and improve the signal‐to‐noise ratio. Han et al. developed a flexible hydrogel made from polyvinyl alcohol and polyvinylpyrrolidone with a modulus similar to tissues. Higher adhesion and lower interfacial impedance were achieved by incorporating nanoparticles produced by the oxidation of polydopamine into the hydrogel. The hydrogel is able to convert prefrontal electroencephalogram signals into sustained attention levels with an accuracy of up to 91.5% (compared to 66.5% for commercial gel electrodes).^[^ ^116^ ^]^ Since Young's modulus of hydrogels can be freely adjusted by changing the chemical composition of the polymer, cross‐linking density, and water content, hydrogel interface electrodes can serve multiple functional modes.^[^ ^117^ ^]^ Su et al. designed an injectable hydrogel with instantaneous bidirectional conductivity that demonstrated excellent performance in the repair of the sciatic nerve in rats.^[^ ^118^ ^]^ Drug delivery based on the 3D network structure of hydrogels is another versatile application of hydrogel interfaces. The network structure of hydrogels can be formed through physical or chemical cross‐linking, allowing hydrogels to encapsulate and store drug molecules. These drugs can be delivered to surrounding tissues through swelling diffusion mechanisms, degradation control, or responsive release. In addition, hydrogel electrodes can be customised to achieve specific needs, being designed to promote or inhibit biological activity.^[^ ^119^, ^120^ ^]^

## 4 Integrated Design for ISS

**Figure 5:**
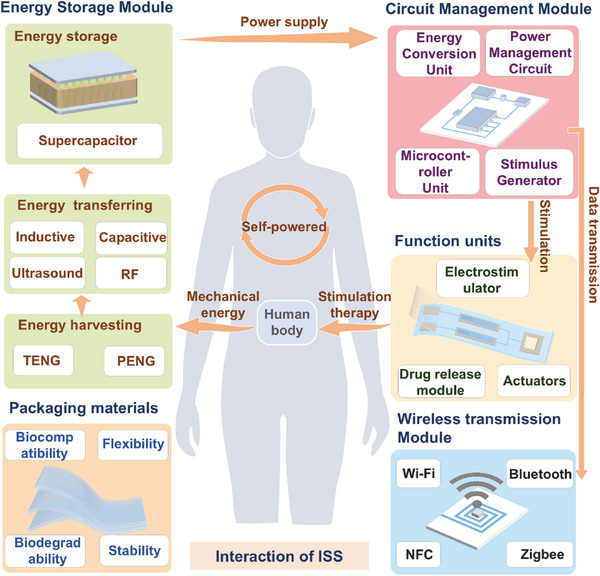
Integrated design of ISS. ISS consists of five modules, mainly including energy storage, circuit management, function module (drug release), packaging, and wireless transmission module.

The integrated development of implantable self‐powered electrical stimulation medical devices represents a multifaceted endeavor that intersects multiple disciplines and witnesses continuous technological innovations. This progression is primarily attributed to the concerted advancements in materials science, biomedical engineering, microelectronics, and energy technologies. Beyond the core components of energy modules and electrode modules, a standalone bioelectronic system can additionally integrate several important elements, including a stable energy storage module, circuit management module, function units, biocompatible packaging materials, and wireless transmission module (**Figure** **5**). Among these, the evolution of supercapacitors as the primary energy storage solution and bioresorbable circuits merit special attention.

### 4.1 Energy Storage

While traditional lithium‐ion batteries possess high energy densities, their toxicity, flammability, and limited lifespans render them unsuitable for long‐term implantation within the human body.^[^ ^121^ ^]^ In ISS systems, supercapacitors have emerged as the preferred energy storage units. Supercapacitors exhibit excellent biocompatibility, minimizing the risk of immune responses or rejection. Additionally, it boasts high power densities, long cycle lives, small volumes, and requires minimal maintenance, thereby fulfilling the energy demands of ISS systems.^[^ ^122^, ^123^ ^]^

The electrode materials for supercapacitors primarily encompass: 1) Carbon‐based materials (e.g., carbon nanotubes, graphene), which are widely utilized due to their high conductivity, large specific surface areas, and superior mechanical properties.^[^ ^124^, ^125^ ^]^ 2) Redox‐active materials (e.g., metal oxides, conductive polymers), which enhance the energy density of supercapacitors through Faradaic charge storage mechanisms.3. Biodegradable materials (e.g., polylactic acid (PLA), polyglycolic acid (PGA)), which gradually degrade within the human body, mitigating long‐term impacts on tissue.^[^ ^126^ ^]^

Regarding electrolytes, aqueous electrolytes are prevalently employed in biocompatible supercapacitors due to their excellent ionic conductivity and biocompatibility. Conversely, organic electrolytes, despite offering higher voltage windows and energy densities, suffer from poor biocompatibility, limiting their adoption in implantable electronic medical devices.^[^ ^127^ ^]^

Electrode materials are a crucial component of supercapacitors.^[^ ^124^ ^]^ He et al. synthesized a series of biocompatible carbon nanotube fibers as electrodes, fabricating novel supercapacitors capable of operating directly in physiological fluids such as phosphate‐buffered saline, serum, and blood.^[^ ^128^ ^]^ These supercapacitors achieved a specific capacitance of 10.4 F cm^−^^3^ or 20.8 F g^−1^, maintaining 98.3% of their initial capacitance after 10 000 cycles in phosphate‐buffered saline. Carbon nanomaterials have emerged as one of the most favored electrodes for energy storage applications, owing to their advantages including high conductivity, stability, porosity, low cost, and ease of processing.^[^ ^129^ ^]^ However, the low capacitance of most carbon‐based materials, which translates to low energy density, has been a limiting factor in practical applications. To overcome this limitation, redox‐active electrode materials have been explored, encompassing metals, metal oxides, metal sulfides, metal nitrides, and various polymers.^[^ ^130^ ^]^ For instance, Li et al. developed a highly biocompatible supercapacitors (B‐SC) using ZnO electrodes and a polymer gel electrolyte.^[^ ^131^ ^]^ This B‐SC features a symmetric hierarchical structure composed of a PLA support substrate, PLA nanopillar arrays, a self‐assembled ZnO nanoporous layer, and a polyvinyl alcohol/phosphate‐buffered saline (PVA/PBS) hydrogel. Its capacitive performance is comparable to other reported superconducting materials and exhibits superior performance in liquid environments. Furthermore, the favorable growth trend of L929 cells on the B‐SC underscores its excellent biocompatibility.

With advancements in medical technology, biodegradable supercapacitors have emerged as a research focus aimed at minimizing the need for secondary surgeries to retrieve devices. Lee et al. reported a fabrication strategy and application of biodegradable micro‐supercapacitors utilizing water‐soluble (i.e., physically transient) metal (W, Fe, and Mo) electrodes, biopolymers, hydrogel electrolytes (agarose gel), and biodegradable poly (lactic‐co‐glycolic acid) substrates. The biodegradable micro‐ supercapacitor featuring Mo interdigitated electrodes and an entirely degradable assembly achieved an areal capacitance of 1.6 mF cm⁻^2^, an energy density of 0.14 µW h cm⁻^2^, and a power density of 1.0 mW cm⁻^2^. These values are comparable to those of non‐transient supercapacitors. The output voltage and capacitance of the biodegradable micro‐ supercapacitor can be tuned by arraying devices in series or parallel connections. The device exhibited stable performance upon immersion in phosphate‐buffered saline (PBS) for a defined period, culminating in complete dissolution.^[^ ^132^ ^]^

Furthermore, Sheng et al. presented a fully biodegradable, high‐performance supercapacitor implant characterized by its lightweight, thin structure, mechanical flexibility, and adjustable degradation duration.^[^ ^133^ ^]^ This supercapacitor employs 2D amorphous molybdenum oxide (MoO_x_) nanosheets as electrodes, which were grown in situ on water‐soluble molybdenum foil using a green electrochemical strategy. The resulting device demonstrates a high areal capacitance of 112.5 mF cm⁻^2^ at 1 mA cm⁻^2^ and an energy density of 15.64 µWh cm⁻^2^.

To accommodate the softness and dynamic changes of human tissues, flexible structural designs are crucial for supercapacitors to enhance their wearability, comfort, and adaptability. Lv et al. fabricated a hydrogel‐based supercapacitor through a straightforward method, where multi‐network conductive electrodes were formed in situ by sequentially crosslinking amine‐reduced graphene oxide and methacrylic anhydride co‐modified sericin (SrMA/A‐rGO) with four‐arm polyethylene glycol succinimidyl carbonate and polyethylene glycol acrylate.^[^ ^134^ ^]^ This supercapacitor exhibited an equivalent series resistance of 21 Ω cm⁻^2^, a volumetric energy density of 26.0 µW cm⁻^2^, and a high specific capacitance retention rate (exceeding 76.4%) after long‐term charge–discharge cycles. As a direct power source, it successfully restarted a stopped heart through electrical stimulation. Furthermore, Sheng et al. demonstrated biodegradable Zn‐ion hybrid supercapacitors employing molybdenum sulfide (MoS₂) nanosheets as the cathode, an ion‐crosslinked alginate gel as the electrolyte, and zinc foil as the anode, achieving high capacitance (93.5 mF cm⁻^2^) and an output voltage of 1.3 V. The power supply capability of these supercapacitors was successfully demonstrated in the controlled release of drugs.^[^ ^135^ ^]^ To summarize, **Table** **1** compiles the electro stimulatory materials and key chemical parameters of supercapacitors reported in recent literature.

**Table 1:** The electro stimulatory materials and key chemical parameters of supercapacitors.

Positive electrode	Negative electrode	Voltage [V]	Power density [mW cm^−2^]	Energy density [µWh cm^−2^]	Refs.
Oxidized SWCNTs	Oxidized SWCNTs	1.0	500	7.12*10^3^	[136]
MoOx	MoOx	0.8	2.53	15.64	[133]
NAD/BQ/CNT	NAD/BQ/CNT	0.8	446*10^3^	19.81*10^3^	[137]
Zn@PPy	Zn@PPy	1.0	0.38	0.394*10^3^	[138]
PEDOT: PSS/ ferritin/ MWCNT	PEDOT: PSS/ ferritin/ MWCNT	0.8	0.15	0.82	[139]
Gold	Gold	0.8	400	263	[140]
Cu_2_O	Cu(OH)_2_	/	9.5	1.74	[141]
Crystalline tetra‐aniline	Crystalline tetra‐aniline	/	0.82	80.3	[142]
Sericin	reduced graphene oxide	/	0.026	0.014	[134]
Activated carbon	gold	/	0.78	10.86	[143]
Mg	MoO3	/	196	4.70	[144]
MnO2	CNT	/	0.819	84.18	[145]

### 4.2 Circuit Management

In the routine operation of electronic devices, a constant direct current (DC) voltage or specific electrical pulses are fundamental requirements for most systems. However, it is noteworthy that energy harvesting technologies based on PENGs and TENGs inherently produce alternating current (AC) output, often at low frequencies. This AC output characteristic poses significant challenges, including energy supply instability and the inapplicability of low‐frequency, uncontrollable pulse signals directly to specific medical devices such as neural stimulators or cardiac pacemakers.^[^ ^146^ ^]^

To overcome these obstacles, rectification technology is typically employed to convert AC into DC for efficient storage in rechargeable batteries or supercapacitors. Nevertheless, this conversion process inevitably leads to energy loss, with rectifiers typically consuming ≈10% to 15% of the input energy, thereby reducing overall energy efficiency. Given the above analysis, optimizing rectifier design to minimize energy losses during conversion and exploring the application of novel flexible, lightweight, and even biodegradable materials are of paramount importance for enhancing the performance of ISS.

In the innovative development of electronic components, significant breakthroughs have been made in flexible and bendable technologies compared to traditional rigid and flat structures, particularly evident in biomedical applications, especially in the development of brain, neural, and cardiac electrical stimulation devices.^[^ ^147^, ^148^, ^149^ ^]^ Xie and his team have pioneered a minimally invasive interventional strategy as an alternative to traditional craniotomy, designing a centimeter‐scale mesh electrode architecture with a sub‐micron thickness that can be precisely implanted through a syringe needle, significantly enhancing surgical safety and convenience.^[^ ^150^ ^]^ This electrode, with its high porosity, tightly adheres to brain tissue, and its porous design not only imparts extreme flexibility but also achieves an effective bending stiffness exceeding 0.64 × 10^−15^ Nm^2^, successfully recording local field potentials from 13 channels in the brain of anesthetized rats. Another critical advancement is the development of a mechanically adaptive flexible neural interface that utilizes an ultra‐thin polyimide‐based structure and is spirally wound around a nerve, enabling mechanical adaptability to neural tissue, thereby significantly optimizing neural signal recording quality. Experiments have shown that, after securing the neural device with sutures, the spiral band electrode can stably record ≈60 evoked neural signals from the rat's peroneal nerve, validating its immense potential in neuroscientific research.^[^ ^151^, ^152^, ^153^ ^]^

Furthermore, research has focused on continuous heart signal monitoring with high signal‐to‐noise ratios. This device integrates organic electrochemical transistors as sensors and an organic photovoltaic (OPV) power source on an ultra‐flexible parylene substrate.^[^ ^153^ ^]^ Notably, the OPV device employs an innovative nano‐grating structured charge transport layer with a periodicity of 760 nm, significantly enhancing OPV efficiency. The thickness of this OPV component is reduced to just a few microns, achieving a remarkable power density of 11.46 W g^−1^ per unit weight, sufficient for use as a portable power source. This self‐powered system successfully integrates and powers electrochemical transistors for continuous monitoring of cardiac activity in live rats.

Machine learning algorithms are progressively being integrated into the information processing module, particularly in the realm of analyzing physiological signals acquired through self‐driven technological means, encompassing electroencephalogram (EEG) signals, electrocardiogram (ECG) signals, pulse signals, and respiratory signals. These algorithms exhibit formidable capabilities in the intelligent processing and analysis of such data. Initially, it facilitates an accurate assessment of a patient's current health status by comparing the patient's real‐time physiological signals against a comprehensive library of standard signals. This comparative analysis hinges on both sophisticated signal processing techniques and advanced feature extraction methodologies, as well as a robust repository of standard signals serving as a benchmark. Moreover, machine learning algorithms possess the capacity to construct precise predictive models through the meticulous mining and analysis of extensive patient history data and treatment outcome information.

A myriad of absorbable conductive materials has found application in implantable medical devices for electrical stimulation, encompassing polymeric conductors such as polyaniline, polypyrrole, and PEDOT, which undergo gradual degradation within the body.^[^ ^154^ ^]^ Critically, the design of degradable conductors necessitates meticulous control over degradation rates to avert premature functional loss. Notably, composite conductive materials, integrating polymers with inorganic elements, aim to harness the strengths of each component, achieving an optimal balance of these properties. These materials exhibit responsiveness to chemical, thermal, electrical, optical, or mechanical stimuli, and modulating their degradation cycles through external stimuli represents an effective strategy.

Currently, the majority of evaluations on bioabsorbable electronic products are conducted in vitro, simulating bodily fluids, and in vivo, using small animal models. However, the long‐term implications of bioabsorbable devices and their degradation products on the biological environment remain largely unknown.

### 4.3 Drug Release

ISS modulates the function of specific neural or tissue regions by delivering electrical signals through microelectrodes. By integrating a drug release module, this system significantly enhances its therapeutic capabilities, achieving a synergistic effect between electrical stimulation and pharmacological treatment.^[^ ^155^, ^156^, ^157^ ^]^ This combination not only improves the precision and efficiency of treatment but also demonstrates substantial potential in various medical fields, including neurological disorders, cardiovascular diseases, pain management, and contraception.^[^ ^158^ ^]^

Spizzirri et al. made notable contributions by developing electrically responsive hydrogel microspheres for the controlled release of diclofenac sodium salt. By optimizing the content of multi‐walled carbon nanotubes, they significantly enhanced the electrical sensitivity of the microspheres and precisely controlled the drug release process.^[^ ^159^ ^]^ Huang et al., on the other hand, focused on the combination of microneedle electrical stimulation and anti‐inflammatory drugs. They designed an electronic drug delivery system that successfully demonstrated the promotion of drug absorption and muscle regeneration in a rat model of skeletal muscle injury.^[^ ^160^ ^]^ This discovery further validates the synergistic effect of the ISS system during the treatment process. Jeon et al. innovated through materials science by fabricating a nanoporous membrane doped with polypyrrole (PPy) on a porous anodized alumina substrate. This membrane undergoes redox state changes under electrical stimulation, resulting in the modulation of membrane volume and pore size, which enables rapid and efficient drug release. With its extremely short response time (less than 10 s and high drug release throughput, this system offers strong support for emergency medical scenarios such as first aid treatment.^[^ ^161^ ^]^ ISS and its integrated drug release module are poised for widespread application across multiple medical fields.

### 4.4 Packaging Materials

With the advancement of materials science, researchers continue to explore and optimize the properties of encapsulation materials to meet the stringent requirements of implantable electronic devices in complex physiological environments. First, ideal encapsulation materials must possess high biocompatibility to ensure their long‐term presence within the human body without eliciting rejection or toxic reactions. Simultaneously, their biostability is paramount to prevent rapid degradation or adverse changes within the body. Moreover, given the diverse functionalities of implantable electronic devices, encapsulation materials must also exhibit suitable mechanical properties, conductivity, thermal conductivity, and insulation properties to support the devices' normal operation. Notably, research on degradable encapsulation materials is particularly compelling. On the other hand, the emergence of flexible encapsulation materials has infused new vitality into the development of implantable electronic devices. These materials, with their superior flexibility and bendability, can closely conform to the intricate shapes of human tissues, minimizing friction and damage to surrounding tissues.

By combining different types of materials, researchers have achieved complementary and optimized performance. These composite encapsulation materials not only possess advantages unparalleled by single materials but can also be customized according to specific application requirements, satisfying the usage demands of implantable electronic devices in diverse scenarios.

### 4.5 Wireless Network Connection

Enabled by advancements in wireless network connectivity technologies, implantable electronic medical devices have achieved seamless integration with smart terminals such as smartphones and tablets. This connectivity not only empowers patients to access their health data anytime, anywhere but also allows physicians to remotely monitor patients' conditions, enabling timely interventions and treatments. Furthermore, wireless network connectivity facilitates the sharing and analysis of medical data, promoting optimal allocation of medical resources and accelerating scientific research and innovation.

As the Internet of Things (IoT), big data, and artificial intelligence (AI) technologies continue to integrate deeply, medical services are becoming increasingly intelligent and personalized. Implantable electronic medical devices are evolving beyond mere monitoring tools to serve as bridges connecting patients, doctors, healthcare institutions, and research facilities, collectively fostering an efficient, collaborative, and sustainable medical ecosystem. However, the development of wireless network connectivity in implantable electronic medical devices confronts several challenges, including but not limited to data transmission security, privacy protection, and power consumption management. Addressing these challenges necessitates interdisciplinary research efforts and innovative solutions to ensure the safe, secure, and efficient utilization of this technology, ultimately enhancing patient care and advancing medical progress.

## 5 The Application of ISS in Medical Electronics

Electrical signals pervade biological systems, serving as an indispensable cornerstone for numerous physiological activities. Within the realm of neural modulation, electrical stimulation technology enables precise manipulation of electrical pulse transmission, thereby activating or inhibiting neuronal activity in specific brain regions. This, in turn, fosters the restoration and remodeling of brain functions. Additionally, this technology has demonstrated remarkable value in cardiac electrophysiology, where the application of electrical pulses effectively regulates arrhythmias, restoring the heart's normal rhythm.

At the local neural level, electrical stimulation disrupts the conduction pathways of peripheral nerve action potentials, effectively inhibiting pain signals, and offering a novel strategy for pain management. In the fields of tissue engineering and regenerative medicine, electrical stimulation, as a non‐invasive or minimally invasive therapeutic modality, influences cellular migration, proliferation, and differentiation trajectories, thereby accelerating tissue repair processes and facilitating the restoration of damaged tissues toward normal physiological states. This characteristic holds significant implications for the treatment of various tissue injuries.

**Figure 6:**
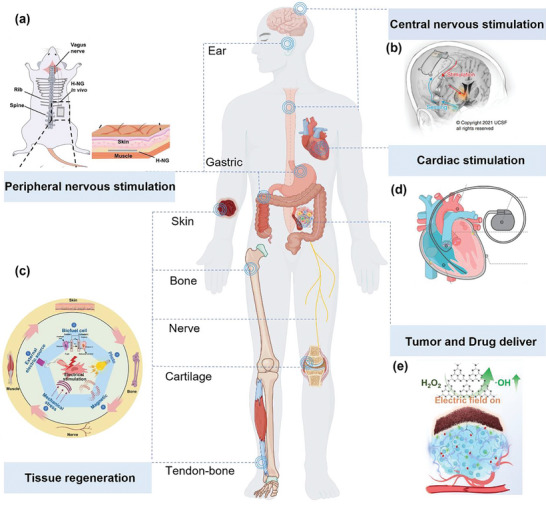
Application of ISS in electrical stimulation therapy. a) ISS used in the vagus nervous stimulation. Hybrid nanogenerators located subcutaneously collect energy and then apply low‐level electrical stimulation to the cervical vagus nerve to treat atrial fibrillation.^[^ ^162^ ^]^ Copyright 2022, Elsevier. b) ISS used in the central nervous stimulation. A closed‐loop implantable Deep Brain Stimulation (DBS) system consists of a biomarker detector and a neurostimulator to treat depression.^[^ ^163^ ^]^ Copyright 2022, Elsevier. c) ISS used in tissue regeneration. The generated electricity by ISS activates various cells and tissues (skin, bone, nerve cartilage, and tendonbone), contributing to the healing and regeneration process.^[^ ^164^ ^]^ Copyright 2024, Springer Nature. d) ISS used in cardiac stimulation.^[^ ^15^ ^]^ Copyright 2020, American Chemical Society. e) ISS used in tumor and drug deliver. Self‐driven electric field stimulation‐promoted cancer catalysis therapy and chemotherapy system based on the triboelectric nanogenerator and implantable nanofibrous patch.^[^ ^165^ ^]^ Copyright 2023, American Chemical Society.

Furthermore, electrical stimulation presents promising prospects in sensory function restoration, particularly in the rehabilitation of auditory and tactile functions. By mimicking natural stimulus signals, it promotes the regeneration and functional reconstruction of damaged neurons. Notably, ISS has emerged as a formidable force in medical electrical stimulation applications due to its unique self‐powering capabilities, exceptional biocompatibility designs, and streamlined manufacturing processes (**Figure** **6**). In recent years, intensive research by academia and industry into ISS systems within the medical electronics landscape has not only broadened their application spectrum but also provided robust technological support for the advancement of cutting‐edge fields such as personalized medicine, remote monitoring, and precision therapy.

### 5.1 Symbiotic Cardiac Pacemakers

The pacemaker is an implantable cardiac electronic medical device used to treat cardiac arrhythmias. The function of the heart to beat is achieved by stimulating the right and left atria and the AV node, thereby triggering contractions through electrical impulses generated by the sinus node. When the body produces irregular signals or abnormalities in the delivery of electrical impulses, arrhythmia results. Thus, pacemakers treat a variety of heart conditions by delivering electrical stimulation similar to that of the sinus node to trigger the heart to beat.^[^ ^166^, ^167^ ^]^ Due to limited battery life, conventional implantable cardiac e‐medicine devices make it difficult to provide long‐term, uninterrupted treatment to patients. Disposable batteries, which have traditionally been used in pacemakers, have become a key constraint for the further development of pacemakers.^[^ ^168^ ^]^ On the one hand, the limited lifespan of disposable batteries makes it difficult to provide long‐term, uninterrupted treatment for patients, which inevitably leads to the risk of secondary surgery. On the other hand, the large size and high rigidity of disposable batteries greatly limit the need for miniaturization and flexibility of pacemakers. Happily, the rise of self‐powered technology effectively solves the many difficulties faced by disposable batteries and brings a new dawn for the development of cardiac pacemakers.^[^ ^169^, ^170^ ^]^

The symbiotic pacemaker (SPM) is a self‐powered cardiac pacemaker whose core feature is the conversion of energy from the beating heart into electrical energy to power the pacemaker.^[^ ^171^ ^]^ The main components of the SPM include an implantable friction electric nanogenerator, an energy management module, and circuitry. Since cardiac pacing requires only microwatt‐level power, the electrical energy converted by the symbiotic pacemaker from the mechanical energy of the beating heart is sufficient to power the cardiac pacing. Inspired by the phenomenon of biosymbiosis, TENG and PENG are prepared by material selection and structural design to enable the self‐powering of the pacemaker. SPM has received much attention from researchers in the past decade, and a number of advances have been made in the field of implantable electronic medical devices.^[^ ^171^, ^172^, ^173^ ^]^

**Figure 7:**
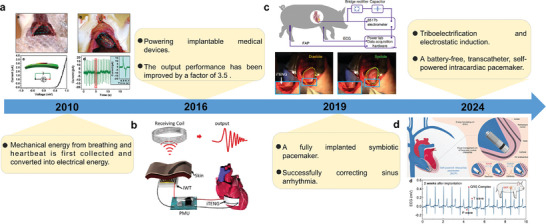
Representative application developments of ISS in pacemakers. a) Nanogenerators were used to harvest energy from the breath and heartbeat of rats.^[^ ^175^ ^]^ Copyright 2010, Wiley‐VCH. b) The iTENG was driven by the heartbeat of an adult pig to enable wireless cardiac monitoring.^[^ ^176^ ^]^ Copyright 2016, American Chemical Society c) Fully symbiotic implantable TENG enabled energy harvesting storage and pacing of the heart.^[^ ^43^ ^]^ Copyright 2019, Springer Nature. d) A battery‐free, transcatheter, self‐powered intracardiac pacemaker based on the coupled effect of triboelectrification and electrostatic induction for the treatment of arrhythmia in large animal models.^[^ ^44^ ^]^ Copyright 2024, Springer Nature.

Since the first proposal and invention of TENGs by Zhonglin Wang et al. in 2006, TENGs have emerged as a viable alternative to traditional disposable batteries by virtue of their self‐powered energy harvesting.^[^ ^174^ ^]^ TENGs are capable of converting tiny mechanical energies (e.g., body movement, vibration, rotation, etc.) into electrical energy, and thus offer a new form of power support for small wireless autonomous devices.^[^ ^12^ ^]^ As a proof‐of‐concept, Li et al. first implanted an AC friction nanogenerator into a living organism in 2010, and the implanted TENG was able to convert small mechanical energies, such as heartbeat and respiration, into a voltage of 2–4 mV and a current of 4–30 pA.^[^ ^175^ ^]^ The ability of the TENG to harvest the small mechanical energies of living organisms offers a new strategy for the power supply of implanted electronic devices (**Figure** **7**a).

In order to further improve the energy conversion efficiency and energy output of TENG, researchers have carried out a series of scientific studies. Zheng et al. developed an implantable friction nanogenerator (iTENG), which collects the mechanical energy generated by the heartbeat by attaching it to the heart surface of a living organism.^[^ ^176^ ^]^ Heartbeat‐induced open‐circuit voltage (Voc) of iTENG in adult Yorkshire pigs reach 14 V, with a corresponding short‐circuit current (Isc) of 4–30 pA. Based on this iTENG, the authors developed a self‐powered wireless transmission system (SWTS) that enables the output of heartbeat‐related electrical signals in vivo, verifying the feasibility of this system for real‐time remote cardiac monitoring (Figure 7b).

Based on the success of TENG in harvesting mechanical energy from the beating heart, Ouyang et al. further developed a self‐powered symbiotic pacemaker.^[^ ^43^ ^]^ This symbiotic pacemaker is a milestone work of symbiotic pacemaker by collecting the mechanical energy of heart beat through TENG and supplying electrical energy to the pacemaker. When the device was fully implanted into an adult pig, the TENG induced an output voltage of up to 65.2 V through heart beats and converted energy of up to 0.495 µJ (higher than the endocardial pacing threshold energy of 0.377 µJ) for a single heart beat cycle. Further, the device enables correction of sinus arrhythmia by harvesting energy from the heart beat. The development of the symbiotic pacemaker solved the challenge of the continuous energy supply of the pacemaker, thus effectively avoiding the risk of secondary removal (Figure 7c). In addition, in order to further improve the energy harvesting efficiency of self‐powered electronic devices, researchers have further improved the output performance of TENGs through multimodal association of TENGs. For example, Liu et al. developed a self‐powered intracardiac pacemaker delivered through a catheter by coupling the friction electric effect with the electrostatic induction effect.^[^ ^44^ ^]^ This pacemaker achieved an open‐circuit voltage and short‐circuit current of 6.0 V and 0.2 mA, and endocardial pacing was realized in a porcine heart. The multimodal TENG device further improves energy harvesting efficiency and further advances the development of implantable self‐powered pacemakers (Figure 7d).

In addition, the development of PENG using the piezoelectric effect of the material provides a new research idea for the energy supply of implantable electronic devices. PENG works on the principle that when the piezoelectric material is deformed, the positive and negative charge centers inside it are displaced relative to each other, thus generating a potential difference, and the electrical output is achieved through an external circuit. The energy harvester was able to generate a short‐circuit current of 0.223 mA and an open‐circuit voltage of 8.2 V, further enabling the regulation of heart rate in rats.^[^ ^170^ ^]^ Xu et al. increased the output power of PENG by 46 times by doping ZnO nanoparticles and polycarbon nanotubes in P(VDF‐TrFE) films. Based on this, a cylindrical energy‐harvesting protomer placed inside the heart was developed, enabling the self‐powering of pacemakers.^[^ ^177^ ^]^

### 5.2 ISS in Nervous System

Electrical stimulation of neurons is an effective means of repairing damaged neurons as well as modulating limb activity downstream of the nerves. Electrical stimulation targeting specific areas of the brain can modulate the activity of damaged neurons in the brain and enhance brain function. Thus, electrical stimulation through the brain is a cutting‐edge approach to treating neurological disorders, including Parkinson's disease,^[^ ^178^, ^179^ ^]^ epilepsy,^[^ ^180^ ^]^ depression,^[^ ^181^ ^]^ and tremor.^[^ ^182^ ^]^ Delivering electrical currents to targeted areas of the spinal cord has the potential to restore functions that are impaired due to injury or disease, such as relieving pain and rejuvenating damaged muscles.^[^ ^183^ ^]^ In addition to this, the vagus nerve, which consists of motor and sensory fibers, is an important part of the nerves that regulate the heartbeat, respiration, digestion, and immunity, amongst others, and has an extensive connecting role between the various systems. Therefore, electrical stimulation of the vagus nerve is a potential treatment for a wide range of diseases, including epilepsy, heart failure, anti‐inflammatory and weight loss.

**Figure 8:**
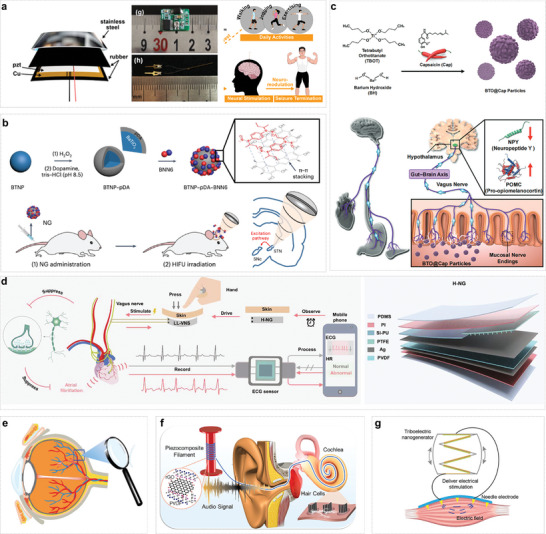
ISS is used in the nerve system. a) Exploded view illustration of a ZnO‐doped PVDF energy harvester used for monitoring epileptic seizures and applying electrical stimulation for therapeutic signals.^[^ ^185^ ^]^ Copyright 2022, Elsevier. b) Modified piezoelectric nanoparticles generate direct current for deep brain stimulation under high‐intensity focused ultrasound.^[^ ^186^ ^]^ Copyright 2023, Springer Nature. c) The self‐powered electrical stimulator targeted sensory nerve endings in the stomach and generated electrical signals through gastric motility, which were transmitted through the brain‐gut axis and ultimately intervened in the hypothalamus to reduce food intake.^[^ ^187^ ^]^ Copyright 2024, Wiley‐VCH. d) A closed‐loop self‐powered LL‐VNS system that can monitor the patient's pulse wave status in real‐time and conduct stimulation impulses automatically during the development of atrial fibrillation.^[^ ^162^ ^]^ Copyright 2022, Elsevier. e) The artificial photoreceptors based on gold nanoparticle‐decorated titania nanowire arrays, for restoration of visual responses in the blind mice with degenerated photoreceptors.^[^ ^188^ ^]^ Copyright 2018, Springer Nature. f) Self‐powered acoustic sensors that respond to sound vibrations to replace cochlear hair cells.^[^ ^189^ ^]^ Copyright 2024, John Wiley and Sons. g) A self‐powered system of a stacked‐layer TENG and a multi‐channel epimysial electrode for rehabilitation and therapeutic purposes in the treatment of muscle dysfunction.^[^ ^12^ ^]^Copyright 2019, Wiley‐VCH.

Thanks to the advantages of an implantable self‐powered electrical stimulation system, which does not require a power supply and has the advantage of continuous and stable electrical stimulation, it has shown great potential for electrical stimulation of neurological disorders.^[^ ^171^, ^184^ ^]^ TENG elicits nerve excitation at thresholds below that of conventional square‐wave electrical stimulation, demonstrating the feasibility of TENG electrical nerve stimulation. In this section, we summarize the application of implantable self‐powered electrical stimulation devices for brain, spinal cord, and vagus nerve‐related electrical stimulation therapy, respectively (**Figure** **8**).

#### 5.2.1 Brain

DBS is widely used in neuroprosthetics and brain‐machine interface, it has a significant modulating effect on neurotransmitter release and synaptic plasticity, DBS can trigger the release of calcium and glial transmitters, promote the dilatation of small arteries, and enhance the local blood flow, so DBS has a more obvious therapeutic effect on improving limb stiffness, bradykinesia and tremor. Hwang et al. developed a high‐performance flexible piezoelectric energy harvesting device for electrical brain stimulation in mice, which uses indium‐modified crystalline Pb(In_1/2_Nb_1/2_)O_3_–Pb(Mg_1/3_Nb_2/3_)O_3_–PbTiO_3_ (PIMNT) films to convert tiny mechanical motions into electricity. Due to the small size of the ferroelectric domains of the ternary PIMNT crystal films, currents of up to 0.57 mA and voltages of up to 11 V could be generated at small deformations and forelimb movements were induced by direct electrical stimulation of the M1 cortex in mice. Zhao et al. reported an electrically stimulated PENG device that stimulates the mouse hippocampus and achieves modulation of neural plasticity in mice, achieving synaptic perceptual plasticity modulation in both states of long‐term potentiation (LTP) and long‐term depression (LTD) by electrical stimulation with PENG,^[^ ^190^ ^]^ providing sufficient data support for implantable self‐powered electrical stimulation device to achieve deep brain electrical stimulation for treatment.

Lin et al. developed a self‐powered electrical brain stimulation system that can monitor and suppress epilepsy.^[^ ^185^ ^]^ Specifically, the system has an energy harvesting unit of piezoelectric lead zirconate titanate to harvest mechanical energy generated by human movement, and PVDF doped with zinc oxide nanostructures as a motion‐sensing sensor to monitor seizures. During a seizure, the data processing center receives the motion‐sensing signal and sends an electrical stimulation treatment signal downstream so that the seizure is suppressed. With the therapeutic effect of this system, the total duration of seizures in mice was effectively reduced by 40–50% (Figure 8a).

Piezoelectric bioactive materials are biomedical materials that generate electrical potentials under mechanical stress. Combining the piezoelectric effect with bioactivity, these materials show great potential for biomedical applications. Piezoelectric bioactive materials are ideal for use in electrical stimulation devices because it does not require complex energy harvesting, transmission, and electrode modules, and the material itself acts as the main body of energy conversion while also generating effective electrical signals directly in the vicinity of the material. Kim et al. developed a piezoelectric nanoparticle for non‐invasive, in situ injection for DBS.^[^ ^190^ ^]^ Nitric oxide (NO) breaks the blood‐brain barrier and allows the nanoparticles to enter the brain parenchyma, followed by ultrasound stimulation of the piezoelectric nanoparticles generates electrical signals that stimulate the release of dopamine from dopaminergic neuron‐like cells, which significantly slows down the symptoms of Parkinson's disease in mice by ultrasound treatment (Figure 8b).

#### 5.2.2 Spinal Cord

Spinal cord electrical stimulation refers to the therapeutic effect of activating dorsal column axons and initiating action potentials by epidural electrical stimulation near the dorsal columns of the spinal cord through specific electrodes, thereby relieving pain or restoring impaired function due to injury or disease. Lu et al. developed a biomimetic Z‐type TENG, which was placed at the elbow joint of the rat and produced a voltage output of 15 V.^[^ ^191^ ^]^ The output remained stable under more than 14,000 cycle tests. In the treatment of rats with motor impairment, the electrical stimulation generated by the device was used for both epidural stimulation of the spinal cord and sciatic nerve stimulation in rats, thus developing a sensory‐motor coupled electrical stimulation modality (SMCS). This stimulation modality promotes motor and sensory tract regeneration and axonal neogenesis more significantly than spinal cord electrical stimulation alone.

Wei et al. proposed a therapeutic method based on the TENG for electrical stimulation by herbal acupuncture therapy, which promotes the repair of the nervous system and motor function after spinal cord injury in rats.^[^ ^192^ ^]^ In this work, the soft‐contact FP‐TENG generated bidirectional effective currents acting on two effective acupoints, Dazhui, GV14, and Mingmen GV4, and the continuous controllable currents inhibited the activation of astrocytes at the lesion site and enhanced the survival of neurons in the ventral horn.

In conclusion, electrical stimulation of the brain and spinal cord requires not only solving the power supply problem, but also well‐designed electrode arrays, precise stimulation positions, and stable and controllable electrical signal outputs are not negligible priorities in research. Therefore, although electrical stimulation therapy of the central nervous system by means of a self‐powered approach effectively solves the problem of power supply, electrical stimulation of the central nervous system still faces other engineering problems that need to be solved, including electrode flexibility and adhesion, electrode precision, biosafety, and stability and accuracy of electrical signals. Although implantable self‐powered electrical stimulation systems have great potential for development in this area, clinical applications must be evaluated by healthcare professionals with in‐depth knowledge of the patient's specific situation and past history to ensure the applicability of electrical stimulation therapy. Therefore, the therapeutic modalities of implantable self‐powered electrical stimulation systems still need to be collaborated and explored by researchers in multiple fields, such as clinical, electronics, materials, and physics, in order to realise their practical clinical applications.

#### 5.2.3 Vagus Nerve

The vagus nerve is a nerve composed of a mixture of motor and sensory fibers and has extensive connections with various systems of the body. Multiple branches of the vagus nerve exist in the cervical, thoracic, and abdominal regions, through which the nerve branches innervate the cervical and intrathoracic organs as well as most of the abdominal organs. The vagus nerve senses impulses and controls the activity of the cardiac muscle, smooth muscle, and glands through the conduction organs, which allows for the regulation of the systems of circulatory, respiratory, and digestive.^[^ ^193^, ^194^ ^]^

With the advantages of high safety and few side effects, vagus nerve stimulation (VNS) has broad clinical applications in regulating the body's neural activity, organ function, immune response, and limb sensation. The application of VNS can be traced back as far as the 1880 s for the treatment of patients with epilepsy and depression, and it is now approved for marketing by the FDA.^[^ ^195^ ^]^ In addition, the peripheral nervous system (PNS) is a downstream branch connecting to the central nervous system and has a role in regulating organ and muscle function, thus electrical stimulation of peripheral nerves is also considered an effective modality for electrical stimulation therapy.^[^ ^196^ ^]^

Yao et al. developed a VNS stimulation device for electrical stimulation of the stomach based on gastric motility‐driven TENG, which was effective in controlling body weight in rats by means of self‐powered electrical stimulation. Electrical signals stimulated afferent fibers of the rat vagus nerve to reduce food intake, and within 15 days the rats' body weight was 38% lower than that of the control group (Figure 8c). Similarly, Mac et al. prepared a self‐powered electroactive particle (BTO@Cap) by combining BTO piezoelectric particles with capsaicin, which can be orally immobilized at the vagus nerve in the stomach. The self‐powered electroactive particles bind specifically to Cap‐sensitive sensory nerve endings in the gastric mucosa, generating electrical signals during gastric peristalsis. The electrical signals are transmitted through the brain‐gut axis and ultimately affect the hypothalamus' control of the feeding process, resulting in a 15% weight loss in the experimental group of mice in an obesity‐inducing tes.^[^ ^187^ ^]^

In addition, VNS shows great potential for application in the treatment of cardiovascular diseases. Low‐level vagal nerve stimulation (LL‐VNS) has received much attention because it does not reduce sinus rhythm or atrial conduction. LL‐VNS can inhibit the vicious cycle between autonomic nervous system remodeling and atrial fibrillation and has also achieved a series of exciting results in clinical trials.^[^ ^197^ ^]^ Sun et al. developed a closed‐loop self‐powered LL‐VNS system based on a hybrid nanogenerator that can generate a current output of 5–15 µA (peak to peak). With this system, not only the onset of atrial fibrillation was greatly reduced, but also the inflammatory pathways associated with NF‐κB and AP‐1 pathways were effectively inhibited (Figure 8d).^[^ ^162^ ^]^

VNS has also shown some clinical promise in the field of inflammatory therapies, mainly through the activation of the hypothalamic‐pituitary‐adrenal axis and the initiation of the acetylcholine anti‐inflammatory pathway.^[^ ^198^, ^199^ ^]^ In addition, Kevin Tracey proposes that VNS is a major component of the neuro‐immune system connection, and VNS has also been shown to have a significant anti‐inflammatory effect on a variety of inflammatory diseases such as rheumatoid arthritis, Crohn's disease, irritable bowel syndrome, and fibromyalgia.^[^ ^200^ ^]^

In summary, the application and prospect of VNS in the clinic are broad and promising. VNS therapy has been approved by the FDA and has demonstrated significant results in the treatment of a variety of diseases. Specifically, in the field of neurological disorders, electrical stimulation of the VNS has been used for the treatment of diseases such as epilepsy, refractory depression, and Alzheimer's disease. In particular, VNS has shown significant results in the treatment of epilepsy. The electrodes of the VNS device are mounted on the vagus nerve through minimally invasive surgery, and a pulse generator emits a stimulating current to regulate the abnormal discharges of nerve cells in the brain, thus effectively reducing the frequency and extent of seizures and even curing epilepsy. Therefore, the development of VNS opens up a new therapeutic avenue for patients with drug‐refractory epilepsy, thus improving the quality of life of epilepsy patients. In addition, VNS has shown potential in neurological function recovery after stroke. Animal experiments have shown that VNS can reduce the inflammatory response, regulate the permeability of the blood‐brain barrier, and promote angiogenesis and neural axon regeneration, thus achieving the effect of reducing the area of cerebral infarction, alleviating the nerve damage, as well as effectively improving the ability of learning and memory. Although there are still many problems in clinical application, such as the optimal stimulation parameter setting, the prospect of VNS application in the field of neurorehabilitation is worth looking forward to. With the continuous development and improvement of the technology, VNS therapy will bring benefits to more patients.

#### 5.2.4 Other Nerves

In addition to these common types of electrical nerve stimulation, electrical stimulation is also applied to other nerves to achieve treatment of different diseases, such as visual cortex, auditory, muscle, and peripheral nerve electrical stimulation.

The retina is an important light‐sensitive tissue capable of converting light signals into neuroelectric signals through multiple layers of neuronal activity.^[^ ^201^, ^202^ ^]^ When retinal degenerative diseases occur, photoreceptor damage or even loss leads to vision loss.^[^ ^203^ ^]^ Electrical stimulation through the visual cortex has become an effective way to treat vision loss, dating back as far as 1918. Scientists stimulated the occipital cortex of soldiers with occipital injuries thereby eliciting stable dot‐phosphorus phantom vision, and proposed the conclusion that the cortex supported central vision.^[^ ^204^ ^]^ Optoelectronic devices can directly convert light signals into electrical signals, and constructing optoelectronic devices to cause neural depolarization is a direction of retinal prosthesis research. Tang et al. developed an artificial photoreceptor of oriented gold nanoparticle‐modified titanium dioxide (Au‐TiO2) nano‐arrays that can transduce light‐encoded signals in real‐time in connection with the retina in mice.^[^ ^188^ ^]^ The photoreceptors have high spatial and temporal resolution for green, blue, and near‐ultraviolet light, greatly facilitating the recovery of vision in mice without affecting the rest of the retinal circuit (Figure 8e).

Hair cells are the key to the organism's ability to achieve hearing. Hair cells convert the vibratory signals produced by sound into biological signals that are transmitted to auditory nerve cells and further to the brain to produce the sensation of sound.^[^ ^205^, ^206^ ^]^ Currently, the most effective integrated technology for the treatment of neurological hearing loss is the cochlear implant. Sensory neural hearing loss is mostly irreversible due to the non‐renewable nature of hair cells, and the development of cochlear implants that can replace the function of hair cells is a promising direction.

Inorganic piezoelectric acoustic transducers based on PZT were first developed, but the brittleness and poor biocompatibility of PZT led to difficulties in its application in cochlear implants.^[^ ^206^ ^]^ Saadatzi et al. fabricated sound transducers similar in size to those of the cochlear implant using flexible PVDF, polydimethylsiloxane, and gold electrodes. Although the transducer can achieve sound signal conversion, its electrical output performance needs to be further improved.^[^ ^207^ ^]^ Composite piezoelectric materials have also been developed to enhance piezoelectric properties by mixing other materials with PVDF, such as barium titanate particles, carbon nanotubes, gold nanoparticles, and graphene oxide.^[^ ^208^, ^209^, ^210^ ^]^ In addition to piezoelectric output performance, frequency recognition of sound is a key performance of cochlear implants.^[^ ^211^, ^212^ ^]^ The researchers have developed a structure of gold nanowires vertically aligned on a flexible polymer, which enables the detection of high‐frequency sound vibrations.^[^ ^213^ ^]^ Mokhtari et al. developed a self‐powered acoustic sensor including poly(vinylidene fluoride), poly(vinylidene fluoride)/barium titanate, and poly(vinylidene fluoride)/reduced graphene oxide piezoelectric nanofilaments.^[^ ^189^ ^]^ The transducer is capable of responding to frequency sounds from 50–1000 Hz with an acoustic‐electrical conversion efficiency of 3.25%, has a high sensitivity of 117.5 mV, and good biocompatibility, which further facilitates the development of cochlear implantation (Figure 8f).

Electrical muscle stimulation has been used as a common clinical treatment for muscle injury to prevent and reverse the progression of muscle atrophy as well as to promote recovery of muscle function.^[^ ^214^, ^215^ ^]^ Unlike the stimulation of neurons, electrical muscle stimulation usually requires stimulation currents on the order of milliamperes, and the main factors affecting the effectiveness of the stimulation are the intensity and waveform of the electrical stimulation.^[^ ^216^ ^]^ Wang et al. used stacked multilayers of TENG to directly activate rat's tibias anterior muscles and further explored electrode adaptation at different spatial locations to optimise stimulation efficiency.^[^ ^12^ ^]^ It was found that the TENG with a long pulse width and low current amplitude had very desirable force output stability (Figure 8g).

Data from previous clinical trials have demonstrated that prolonged intermittent electrical stimulation of the oesophageal sphincter restores its normal shielding function, thereby slowing the symptoms of gastro‐oesophageal reflux.^[^ ^217^ ^]^ Zhang et al. developed an implantable device for electrical stimulation of the lower oesophageal sphincter, which actively augments the muscle to increase oesophageal closing pressure without affecting its normal relaxation. This device consists of a nickel‐titanium alloy oesophageal stent as mechanical support and liquid metal (LM) as radio transmission, along with integrated microneedles to achieve transmucosal electrical stimulation. The scaffold was validated in an adult porcine model to demonstrate the efficacy and safety of the E‐Stent in providing electrical stimulation.^[^ ^218^ ^]^ Unlike electrical stimulation that enhances muscle activity, Liu et al. prepared an artificial muscle for oculomotor paralysis disease using a self‐powered system.^[^ ^219^ ^]^ A self‐repairing polydimethylsiloxane based on reversible imine and hydrogen bonds was used as a flexible electrode, and a high‐output TENG was used as an energy supply to develop an extraocular muscle‐like brake that allows directional movement.

Although some progress has been made in muscle electrical stimulation, the mechanism of action of muscle electrical stimulation in sports rehabilitation is still unclear, it can promote muscle recovery in the short term but the long‐term effect is still uncertain. Moreover, the artificial muscle response induced by electrical stimulation is different from the physiological muscle response, which is more prone to fatigue. Therefore, a clear mechanism, the exploration of effective electrical stimulation signals, and the targeting of electrical stimulation to muscles are all directions for future exploration of electrical stimulation muscle therapy.

### 5.3 ISS in Tissue Regeneration

**Figure 9:**
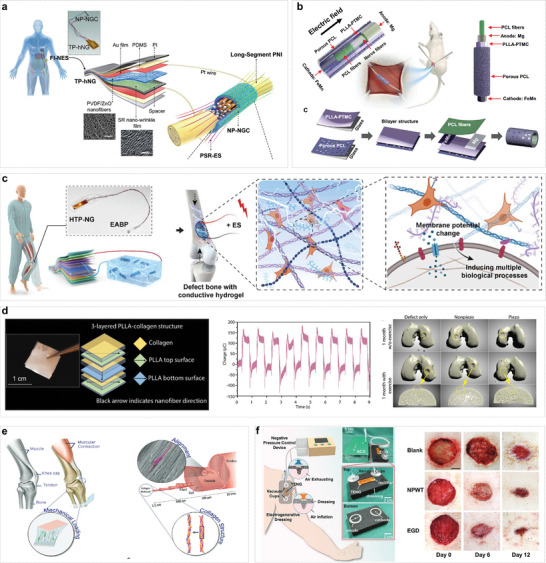
ISS is used in tissue regeneration. a) A physiologically self‐regulated electrical signal is generated by integrating a novel tribo/piezoelectric hybrid nanogenerator with a nanoporous nerve guide to constructing a fully implantable neural electrical stimulation (FI‐NES) system.^[^ ^228^ ^]^ Copyright 2021, Wiley‐VCH. b) A fully biodegradable, self‐electrifying, and miniaturised device which can provide both structural guidance and electrical cues for peripheral nerve regeneration.^[^ ^229^ ^]^ Copyright 2020, AAAS. c) The fully implantable bone defect electrical stimulation (BD‐ES) system combines a hybrid tribo/piezoelectric nanogenerator to provide biphasic electric pulses.^[^ ^230^ ^]^ Copyright 2024, AAAS. d) Exercise‐induced piezoelectric scaffold for cartilage regeneration in rabbits.^[^ ^232^ ^]^ Copyright 2022, AAAS. e) A piezoelectric collagen‐analogue scaffold comprised of aligned nanoscale fibers made of the ferroelectric material poly(vinylidene fluoride‐co‐trifluoroethylene) for tendon tissue regeneration.^[^ ^233^ ^]^ Copyright 2021, John Wiley and Sons. f) An electrogenerative dressing (EGD) integrated triboelectric nanogenerators with negative‐pressure wound therapy (NPWT) for wound therapy.^[^ ^234^ ^]^ Copyright 2023, John Wiley and Sons.

In the complex regulatory network of the tissue microenvironment, the fine regulatory system composed of physical and chemical signals influences the multidimensional behavior of cells including anchoring, migration, proliferation, differentiation, and even apoptosis.^[^ ^220^, ^221^, ^222^ ^]^ Among them, electrical stimulation, as a non‐invasive physical intervention, has been widely recognized by the scientific community as one of the key factors in regulating cellular functions.^[^ ^223^ ^]^ Specifically, the electroactive environment can significantly direct the spatial arrangement of cells, promote cell proliferation, and induce specific cell differentiation patterns. Therefore, electrical stimulation treatment modalities have great potential to promote tissue healing and regeneration during the natural repair process of the organism.^[^ ^224^, ^225^, ^226^, ^227^ ^]^ Mechanistically, electrical stimulation has been shown to trigger intra‐ and extracellular ion flow by activating ion channels on the cell membrane, which in turn triggers the remodeling and dynamic adjustment of the cytoskeleton. Electrical stimulation ultimately promotes the migratory capacity of cells by affecting a series of intracellular events.^[^ ^228^ ^]^ Studies have shown that well‐designed electrical stimulation parameters can precisely regulate different cell types. For example, in the osteoblast system, appropriate electrical stimulation can significantly promote cell proliferation and differentiation, which is important for bone tissue regeneration. Similarly, in the field of stem cells, specific patterns of electrical signals can guide the differentiation of stem cells toward the neural spectrum, providing new perspectives for the treatment of neurodegenerative diseases and the regeneration of neural tissues.^[^ ^229^, ^230^ ^]^ In conclusion, electrical signals, as an efficient and low‐cost biophysical modulation means, show a broad application prospect in the field of tissue engineering and regenerative medicine.^[^ ^231^ ^]^ By precisely modulating electrical stimulation parameters, scientists are able to achieve fine‐grained manipulation of cellular behavior, opening up new pathways for the intervention and treatment of a wide range of biological diseases. In this section, we summarize the applications of implantable self‐powered electrical stimulation medical devices in tissue regeneration, including but not limited to the regenerative repair of nerve, bone, cartilage, and skin (**Figure** **9**).

#### 5.3.1 Nerve

Severe nerve damage can lead to loss of sensation or impairment of motor function, which has a huge impact on the patient's life.^[^ ^235^, ^236^ ^]^ Severe neurological injuries are often treated by interventional autograft surgery, however, autografts face the risk of limited donors and nerve mismatch.^[^ ^237^ ^]^ Artificial neural scaffolds are an effective way to solve the autografting challenge, in which electrical stimulation via artificial neural scaffolds is a novel therapeutic means to achieve nerve regeneration.^[^ ^238^, ^239^, ^240^ ^]^ Similar to electrical stimulation for the therapy of neurological diseases, nerve cells, as electrosensitive cells, have cellular activities that are directly regulated by electrical signals. In the repair of nerve injury, electrical stimulation can accelerate the recovery of nerve function by stimulating neuronal differentiation, maturation, and axonal extension. Researchers have found that AC or DC electric fields not only promote neuronal differentiation of PC12 cells and synaptic growth of dorsal root ganglion neurons but also accelerate the recovery of sciatic and facial nerve function.^[^ ^236^, ^241^, ^242^, ^243^ ^]^ For implantable electrical stimulation devices, the cumbersome percutaneous external wiring directly leads to problems such as limiting the treatment time, restricted patient activities, local infections, etc. Self‐energy‐supplied electrical stimulation devices are able to convert various energies into electrical signals for treatment in vivo avoiding the problem of external wiring.^[^ ^244^ ^]^

Nanogenerators capable of harvesting tiny mechanical energy and converting it into electrical energy show great potential in electrically stimulating nerve regeneration.^[^ ^245^, ^246^ ^]^ Wu et al. developed a piezoelectric polymer scaffold based on zinc oxide nanoparticles, which was effective in treating nerve injury and promoting vascular growth factor expression and blood cell proliferation under ultrasound.^[^ ^247^ ^]^ Further, hybrid modes based on friction electric and piezoelectric nanogenerators were developed for electrical stimulation of nerve regeneration. Jin et al. developed an implantable electrical nerve stimulation device with physiological adaptations by a novel friction/piezoelectric hybrid nanogenerator (TP‐hNG) combined with a multifunctional nonporous nerve catheter (NP‐NGC).^[^ ^228^ ^]^ The device generates electrical signals with extended peak widths driven by respiratory movements subcutaneously in the thoracic cavity and induces migration, proliferation, and myelination of regenerating nerve fibers in Schwann cells via electrical signals. After 3 months of treatment, the device achieved therapeutic efficacy comparable to autografts in an animal model of peripheral nerve injury (Figure 9a).

Similarly, researchers have undertaken numerous efforts in electrical stimulation of nerve regeneration, for example, wirelessly powered cuff electrodes have been proposed to promote sciatic nerve repair,^[^ ^248^ ^]^ non‐degradable self‐powered scaffolds based on glucose fuel cells have been shown to improve axonal growth, bioabsorbable radio‐stimulators coupled via inductive coupling have been realized to enhance regeneration of transected nerves.^[^ ^249^ ^]^ Electrical stimulation has also been shown to promote axonal growth via cyclic adenosine monophosphate (cAMP), release of neurotrophic factors, and proliferation of haemangiocytes.^[^ ^250^ ^]^

In clinical practice, various factors should be considered such as the guidance and degradability of the scaffold, the supply of neurotrophic factors, and the reduction of inflammation, and this combined therapeutic strategy is the most promising approach to improve the accuracy and efficacy of neural regeneration. Wu et al. developed a new methodology based on potassium sodium niobate (KNN) nanowires, poly(lactic acid) (PLLA), and poly(3‐hydroxybutyric acid)‐co‐3‐hydroxyvalerate (PHBV), PLLA or PCL film as encapsulation layer, as well as Mg electrodes and Mo wires in a biodegradable piezoelectric electrical stimulator, which significantly facilitated the regeneration of rat sciatic nerves.^[^ ^251^ ^]^ Wang et al. developed an integrated self‐powered catheterization device for regenerative therapy of rat sciatic nerves, with the electrical signal coming from an absorbable protocell made of thin‐film magnesium and ferromanganese alloy electrodes. The device coupled the protocells and the nerve conduit to provide continuous electrical stimulation while guiding cell growth, which promoted the proliferation of haemangioblasts and the secretion of neurotrophic factors and accelerated the regeneration of the sciatic nerve and the restoration of motor function (Figure 9b).^[^ ^229^ ^]^ Recently, conductive hydrogels that can generate electrical signals in situ based on capacitive coupling effect neural scaffolds were successfully developed to enhance functional recovery by promoting myelin regeneration, accelerating axonal regeneration, and promoting endogenous neural stem cell differentiation.^[^ ^252^ ^]^

#### 5.3.2 Bone

Bone is one of the few tissues in the human body that can regenerate, not only supporting the body but also protecting vital organs. Injury to bone tissue immediately initiates a cellular cascade reaction that promotes a bone‐healing environment and rebuilds bone structure and function.^[^ ^253^ ^]^ In some special cases, such as large infections, tumours, or severe trauma resulting in bone defects can lead to delayed or non‐healing of the bone, causing a huge financial and emotional burden to the patients. It is therefore necessary to find a means that can effectively promote bone regeneration and remodeling. In 1954, Japanese scientist Yasuda first reported that bone has piezoelectricity, and later research further confirmed that the piezoelectricity of bone does not come from living cells, but from collagen fibers in bone.^[^ ^254^ ^]^ Collagen fibers consist of three peptide chains intertwined to form a triple helix structure, which is the direct reason for their piezoelectric properties. Polypeptide chains are formed by connecting amino acids through dehydration and condensation to form peptide bonds. The positive and negative charge centers of peptide bonds do not coincide, specifically, the amino end points to the electric dipole at the carboxyl end. In addition, the hydrogen bonds between amino groups and carboxyl groups are arranged according to the helical structure, so the original dipoles are repeatedly arranged along the helical structure in a unidirectional way, resulting in the permanent polarization of collagen fibers showing piezoelectricity.^[^ ^255^, ^256^ ^]^ The piezoelectric coefficient of bone is equal in size on *d*_14_ and *d*_25_ with opposite signs:*d*_14_ = 0.2 pC/N. When an adult walks, the voltage on the tibia will be produced over 300 µV. The flexoelectricity effect in biological tissues may also be an interesting research direction.^[^ ^256^ ^]^ Because of the piezoelectricity of bone, bone will produce electronegativity when it is subjected to mechanical stress. These negative charges directly promote the proliferation and differentiation of osteoblasts, thus promoting bone regeneration.^[^ ^257^ ^]^ Moreover, the report shows that the electronegativity of the fracture site is stronger than that of the normal bone tissue. These negative charges promote osteogenic activity and contribute to the matrix mineralization of the fracture site. Therefore, electrical signal plays an important role in the repair of bone tissue damage. For many years, people have tried to influence the effect of bone repair by simulating this electric field environment, in which self‐powered implantable electrostimulation devices provided excellent strategies.

The direct application of current or electric field to the area of bone damage via electrodes is a commonly used tool in electrical stimulation intervention therapy. In addition, electrical stimulation therapy has shown excellent results in the treatment of osteomyelitis, in which electrical stimulation modulates the immune microenvironment through the polarization of macrophages.^[^ ^257^ ^]^ Fork‐finger electrodes have been designed to provide an electric field to osteoblasts, Tian et al. used a TENG chain connected to a fork‐finger electrode to provide an electric field stimulation to MC3T3‐E1 cells, resulting in a significant increase in intracellular Ca^2+^ levels and faster differentiation toward osteogenesis after surface electrical stimulation.^[^ ^258^ ^]^ Further, Yao et al. prepared an implantable resorbable fracture electrical stimulation device capable of generating bi‐directional pulsed electricity in response to body movements by combining a flexible TENG with a pair of dressing electrodes. The flexible generator was designed in an arrayed shape to fit various irregular tissue surfaces, and in a rat fracture model, the experimental group achieved the healing effect of the control group at 10 weeks post‐surgery in only 6 weeks. To increase the electrical output power, the poly(lactic‐co‐glycolic acid) (PLGA) surface was machined with tiny pyramidal structures, and the constituent polymer PLGA and electrode Mg metal electrodes in the device were bioabsorbable, eliminating the need for secondary surgery.^[^ ^259^ ^]^ Wang et al. have used the TENG technology to develop an electrical pulse stimulator (BD‐ES) that eliminates the need for circuitry and batteries, solving the problems of weight, volume, and the necessary rigid encapsulation layer. The fully implantable bone defect BD‐ES incorporates a hybrid three‐phase/piezoelectric nanogenerator that delivers biphasic electrical pulses in response to rehabilitative exercise (Figure 9c).^[^ ^230^ ^]^

Similarly, electrical stimulation therapy plays an equally important role in the regeneration of tissues such as cartilage, tendons, rotator cuffs, etc. Liu et al. developed a biodegradable piezoelectric PLLA electrical stimulation scaffold for cartilage regeneration in the joints that was driven by joint loading. The scaffold generated a controlled charge during force application to promote cell migration and recruitment and induced the secretion of endogenous TGF‐β1 via the calcium ion signaling pathway which in turn facilitated the process of cartilage regeneration (Figure 9d).^[^ ^232^ ^]^ Fernandez‐Yague et al. prepared piezoelectric fiber scaffolds to regulate tendon cell activity. P(VDF‐TrFE) piezoelectric scaffolds with collagen‐like piezoelectric properties generate electrical signals during movement promoting the tendon regeneration process by modulating cellular ion channels (Figure 9e).^[^ ^233^ ^]^

#### 5.3.3 Skin

The presence of electric currents at wound sites has been documented for over a century, with this electrical signaling playing a pivotal role in guiding the migration of electrically active key cells such as epithelial cells during wound re‐epithelialization.^[^ ^260^ ^]^ The direct guidance of epithelial cell migration by direct current (DC) sources has also been demonstrated.^[^ ^78^, ^261^, ^262^ ^]^ The electrical potential at wounds originates from the transepithelial potential (TEP) of the normal epidermis. Due to the asymmetric distribution of ion channels within epithelial cells, anions (Cl^−^) are transported to the apical side of the epidermis, while cations (Na^+^) are transported to the basal side, thereby generating the transepithelial potential.^[^ ^263^ ^]^ At the site of wounding, a lateral electric field emerges with the wound margins acting as anodes and the center of the wound as cathodes. A reduction in the electric field at wound sites has been shown to delay the wound‐healing process, which has been validated in some metabolic disease models.^[^ ^261^ ^]^

Self‐powered systems that mimic endogenous electric fields at wound sites represent an effective strategy for promoting wound healing. As shown in Fig, Luo et al. designed an electrically regenerative dressing (EGD) that integrates wound negative pressure drainage devices with TENGs. In a closed wound environment, the negative pressure drives the integrated TENG to convert mechanical energy generated by dressing contraction and relaxation into electrical energy, forming a compensatory electric field for the wound. In an adult pig wound model, the EGD enhanced wound repair quality by reconstructing mature epithelial microstructures and ordered extracellular matrices, while also reducing scar formation (Figure 9f).^[^ ^234^ ^]^ Jeong et al. developed an ionically conductive triboelectric nanogenerator patch, where ionically conductive and stretchable organic gel fibers serve both as stretchable conductors and wound dressings, providing a uniform and symmetrical electric field to the wound. At the cellular level, the device accelerated the migration, proliferation of fibroblasts and dermal cells, and secretion of angiogenic factors in diabetic patients, with its wound‐healing effects validated in animal models.^[^ ^264^ ^]^

Distinct from mimicking endogenous electric fields, electrical signals have also been employed as an effective means of achieving cellular transfection. Xiao et al. developed a degradable electro‐transfection‐assisted scaffold for healing interfaces (E‐TASHI) for wound repair.^[^ ^265^ ^]^ E‐TASHI comprises two modules: a plasmid electro‐transfection module and a cellular scaffold module. The plasmid electro‐transfection module initiates a pulsed electric field within a short period to achieve electroporation of cells at the wound edge, further delivering plasmids that promote cell proliferation and migration into the cells, accelerating their rapid proliferation and migration into the internal pores of the scaffold module. The cellular scaffold, consisting of a porous structure made of biodegradable hydrogels, provides a microenvironment and mechanical support for cell growth and migration and can degrade into amino acids for biological utilization.

Electrical stimulation plays a crucial role in wound healing. Looking ahead, the integration of electrical stimulation therapy with drug delivery, wound dressings, negative pressure drainage, and other modalities will form a more effective comprehensive treatment strategy to further enhance wound healing outcomes. Additionally, for specific wound types such as those in diabetic patients, infected wounds, and elderly individuals, we must delve deeper into the mechanisms of electrical stimulation under these specific conditions to ensure the effectiveness and safety of its therapeutic application.

#### 5.3.4 Drug Delivery and Tumour Therapy

Self‐powered electrostimulatory drug delivery systems (DDS) have emerged as a cutting‐edge frontier in modern medical technology, achieving groundbreaking advancements in promoting personalized and precision medicine. By ingeniously harnessing electrical signals as the medium for regulating drug release, these systems meticulously manipulate the release rate and pattern of drugs at target sites, thereby maximizing therapeutic efficacy and minimizing adverse effects. DDS not only streamlines the treatment process, enhancing patient convenience but also personalizes and refines drug delivery, paving new avenues for tackling multifarious diseases. Electrostimulation drug delivery techniques can generally be categorized into several mechanisms. First, by increasing cellular membrane permeability via voltage, this method utilizes electrostimulation to alter the membrane potential and molecular structure, thereby facilitating the rapid and abundant penetration of drug molecules across the cellular membrane, thus enhancing drug absorption efficiency and utilization. Second, the electrochemical effect refers to the release of drug molecules driven by electric field forces under the action of an electric field, enabling drug delivery. Furthermore, electroporation of cells employs electrostimulation to induce the formation of microchannels or pores on the cellular membrane, serving as direct pathways for drug molecules to enter the cell interior, bypassing the membrane barrier. In the context of electrostimulation for tumor treatment, this technique not only augments the cytotoxic effect of drugs on tumor cells in combination with electrostimulation but also directly kills tumor cells through the electroporation process itself.

**Figure 10:**
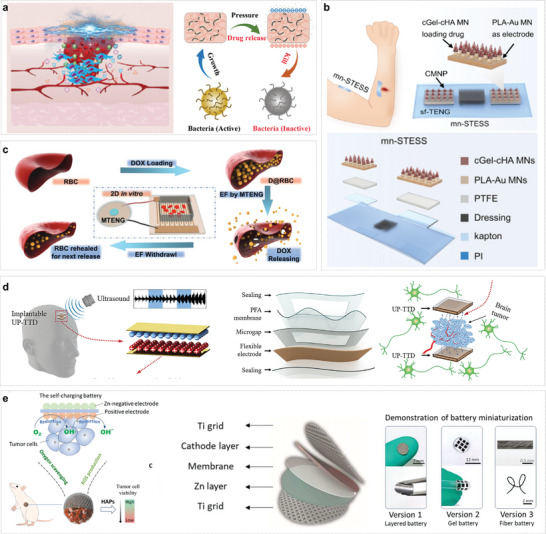
ISS is used in drug release and tumour therapy. a) Aself‐powered wound dressing based on “LOCK‐ON/OFF” EF‐drive drug release combined ES for infection wound repair, which used PVDF as a self‐powered system and c‐MWCNTs‐VAN as an EF‐driven drug release system.^[^ ^266^ ^]^ Copyright 2024, John Wiley and Sons. b) A microneedle‐based self‐powered transcutaneous electrical stimulation system (mn‐STESS) for achieving improved epidermal growth factor pharmacodynamics.^[^ ^267^ ^]^ Copyright 2022, Springer Nature. c) A nanogenerator‐controlled drug delivery system (DDS) for use in cancer therapy.^[^ ^268^ ^]^ Copyright 2019, John Wiley and Sons. d) An implantable ultrasound‐powered tumor treating device (UP‐TTD) that electromagnetically disrupts the rapid division of cancer cells without any adverse effects on normal neurons.^[^ ^269^ ^]^ Copyright 2022, AAAS. e) An implantable self‐charging battery that can regulate tumor microenvironment persistently by the well‐designed electrode redox reaction.^[^ ^267^ ^]^ Copyright 2023, AAAS.

As shown in **Figure** **10**a, researchers have innovatively employed PVDF as the core of a self‐powered system, coupled with vancomycin‐loaded carboxylated multi‐walled carbon nanotubes (c‐MWCNTs‐VAN), to construct an electric field‐responsive drug release system.^[^ ^266^ ^]^ This system autonomously harvests and converts minute mechanical energy from the wound environment into electrical energy, driving on‐demand drug release. Notably, it significantly enhances the penetration and retention of vancomycin at the infection site, effectively accelerating wound healing and mitigating antibiotic overuse and resistance issues. Additionally, Yang et al. explored the potential of electrical stimulation in enhancing epidermal growth factor (EGF)‐mediated wound healing through the development of a microneedle‐based self‐powered transdermal electrostimulation system (mn‐STESS).^[^ ^267^ ^]^ As depicted in Figure 10b, mn‐STESS integrates a sliding‐freestanding triboelectric nanogenerator (sf‐TENG) with composite microneedle patches (CMNP). The sf‐TENG captures and converts mechanical energy generated by skin movements into electricity, while the CMNP serves as both a drug delivery vehicle and an electrostimulation applicator. This system markedly elevates EGF concentrations and retention time at wound sites, fostering tissue regeneration and repair through electrical stimulation, presenting a novel therapeutic approach for accelerated wound healing.

In the context of cancer therapy, self‐powered electrostimulation primarily relies on the synergistic effects of electric fields and drugs, as well as the direct impact of electric fields on tumor cells. As illustrated in Figure 10c, Zhao et al. devised a DDS based on a novel magnetic triboelectric nanogenerator (MTENG) for cancer treatment.^[^ ^268^ ^]^ MTENG boasts efficient energy conversion capabilities, stably generating electrical stimulation signals both in vitro and in vivo. Its unique nanostructured design enables precise control over the release of the anticancer drug doxorubicin (DOX). Under MTENG‐induced electrical stimulation, DOX release rates surge, achieving potent cancer cell eradication at lower doses, drastically reducing the toxic side effects associated with chemotherapy and offering renewed hope for cancer patients.

Furthermore, the application of self‐powered electrostimulation in cancer therapy is grounded in its biological effects on tumor cells and surrounding tissues. Electrical stimulation disrupts tumor cell membrane potentials, induces apoptosis, and enhances drug permeability and efficacy. Yang et al. introduced an implantable ultrasonic tumor treatment device (UP‐TTD) that exerts no adverse effects on normal neurons, safely inhibiting brain cancer recurrence (Figure 10d).^[^ ^269^ ^]^ Both in vitro and in vivo experiments confirmed UP‐TTD's remarkable therapeutic performance, inhibiting clinical tumor growth by ≈58% and reducing cancer area in tumor‐bearing rats by roughly 78%. As shown in Figure 10e, Huang et al. demonstrated an implantable self‐charging battery capable of continuously modulating the tumor microenvironment through orchestrated electrode redox reactions.^[^ ^270^ ^]^ Comprising biocompatible polyimide electrodes and zinc electrodes, this battery consumes oxygen during discharge/self‐recharge cycles, regulating hypoxia levels in the tumor microenvironment. Oxygen reduction within the battery generates reactive oxygen species, conferring 100% prevention against tumor formation.

In conclusion, the field of self‐powered electro stimulatory drug delivery and cancer therapy presents a promising landscape. By achieving energy autonomy and simplifying treatment protocols, these systems significantly enhance therapeutic outcomes through precise drug release and electrostimulation. With the continuous advancement of nanomaterials, biocompatible technologies, and intelligent control systems, the ISS system will become more efficient and safer in clinical applications, providing patients with personalized and precise treatment plans. We summarized the applications of ISS in medical electronics in **Table** **2**. Interdisciplinary collaboration will further advance this field and expand its applications in disease prevention, early diagnosis, and integrated treatment.

**Table 2:** The applications of ISS in medical electronics.

Biological applications	Electrical properties	Effect	Energy harvesting	Refs.
Cardiac Pacemaker	8.2 V, 0.223 mA	Charged batteries and stimulated the heart	PENG	[170]
	14 V, 5 µA	Real‐time wireless cardiac monitoring	TENG	[176]
	3.22 ± 0.24 V	Harvested energy from the heartbeat.	PENG	[177]
	65.2 V, 0.495 µJ	Achieved cardiac pacing and sinus arrhythmia correction	TENG	[43]
	6.0 V, 0.2 µA	Maintained endocardial pacing function	Triboelectrification and electrostatic induction	[44]
Nerve system	≈28 V, ≈150 µA	Restrained the epilepsy of mice	TENG	[185]
	/	Alleviated the symptoms of the disease	Piezoelectric particles	[186]
	≈5 V	Reducted body weight	Piezoelectric particles	[187]
	14.8 V, 17.8 µA	The duration of atrial fibrillation was significantly reduced by 90%.	Hybrid nanogenerator	[162]
	117.5 mV (Pa·cm^2^)^−1^	The overall acoustoelectric energy conversion efficiency of 3.25%	Piezoelectric nanocomposite filaments	[189]
Tissue regeneration	0.068–0.984 V	Promoted calcium activity, repopulation of Schwann cells, and neurotrophic factors	Galvanic cells	[229]
	35 V, 3.7 µA	The femur completely healed within 6 weeks	Tribo/piezoelectric nanogenerator	[230]
	3.6 V	Improved cartilage and subchondral bone regeneration	PENG	[232]
	0.28–1.21 V	Mmodulated tendon cell biological function and tissue repair processes	Piezoelectric fiber membrane	[233]
	4.7 V, 28 nA	Promoted wound repair	TENG	[234]
Drug release	5 V	A 1.26 fold improvement in wound healing	Piezoelectric fibers	[266]
	20 V, 1 µA	Improved the pharmacodynamics of EGF to aid wound healing	TENG	[267]
	70 V	Increased the release of DOX	TENG	[268]
	1.3–1.5 V cm^−1^	≈78% reduction of cancer area in tumor‐bearing rats	TENG	[269]

## 6 Conclusions and Future Perspectives

Implantable self‐powered electrical stimulation medical devices have emerged as a cutting‐edge medical technology, showcasing immense potential and value across various therapeutic domains. Emerging magnetoelastic technologies also have potential for ISS, such as self‐powered cardiovascular monitoring using permanent magnetic fluids,^[^ ^271^ ^]^ the development of liquid cardiac sensors that are stable in dynamic environments,^[^ ^272^ ^]^ innovation of wearable generators and sensors by combining soft magnetoelastic composites with liquid metal coils,^[^ ^273^ ^]^ and the weaving of high‐performance magnetoelastic soft fibers for biomechanical‐electrical energy conversion.^[^ ^274^ ^]^ In addition, scalable magnetoelastic generator arrays can provide tunable electrical stimulation platforms for biological applications and offer the possibility of personalized medicine.^[^ ^275^, ^276^ ^]^From innovations in power modules, particularly the exploration of TENGs and PENGs as novel energy sources, to the diverse applications of rigid and flexible electrodes within the electrode module, these advancements underscore the positive impact of technological progress on enhancing treatment efficacy and improving patients' quality of life. This technology necessitates not only efficient and stable energy supply but also emphasizes the biocompatibility and flexibility of electrode materials to adapt to complex in vivo environments.

In terms of integrated design, the development of supercapacitors as energy storage modules provided strong support for the continued operation of the International Space Station. In addition, research into biodegradable circuitry and packaging materials ensured the efficiency of energy harvesting while mitigating long‐term risks. The integration of wireless communication technologies and machine learning algorithms enabled remote control and data transmission, providing physicians with real‐time and accurate monitoring tools while offering patients a more convenient treatment experience.

Despite the promising application prospects of ISS in biological treatments, a multitude of challenges continue to persist. First, the long‐term stability and reliability of the power module represent a critical issue. Ensuring the sustained and efficient operation of novel energy sources such as TENG and PENG in the complex and dynamic in vivo environment is an urgent problem to be solved. Second, the selection and design of electrode materials pose numerous challenges, requiring a delicate balance between conductivity, biocompatibility, flexibility, and stability—a focus and difficulty of electrode module research. Third, regarding biological treatment applications, the efficacy and safety of ISS require continuous attention. Particularly in the fields of neurological disease treatment, tissue regeneration engineering, drug delivery, and tumor therapy, the mechanisms of action and optimal treatment strategies of ISS remain to be further explored and optimized. Finally, the development of personalized treatment plans, precise regulation of electrical stimulation parameters, and long‐term evaluation of treatment outcomes are all vital areas for future research.

Moreover, issues such as energy management, data transmission security, privacy protection, and power consumption management within the systematic design cannot be overlooked. With the deep integration of technologies like the Internet of Things, big data, and artificial intelligence, ISS are gradually becoming integral parts of the medical ecosystem. Consequently, ensuring efficient system interoperability and data security will be a crucial research direction in the future.

In conclusion, implantable self‐powered electrical stimulation medical devices, as an emerging medical technology, are driving innovative developments in the medical field while confronting numerous challenges. Through sustained research and technological innovation, optimizing power sources, electrodes, systematic designs, and treatment strategies, we can overcome these challenges and achieve widespread application of ISS in various therapeutic domains, thereby making contributions to human health.

## Conflict of Interest

The authors declare no conflict of interest.
